# Novel mouse monoclonal antibodies specifically recognizing β-(1→3)-D-glucan antigen

**DOI:** 10.1371/journal.pone.0215535

**Published:** 2019-04-25

**Authors:** Andrey L. Matveev, Vadim B. Krylov, Yana A. Khlusevich, Ivan K. Baykov, Dmitry V. Yashunsky, Ljudmila A. Emelyanova, Yury E. Tsvetkov, Alexander A. Karelin, Alevtina V. Bardashova, Sarah S. W. Wong, Vishukumar Aimanianda, Jean-Paul Latgé, Nina V. Tikunova, Nikolay E. Nifantiev

**Affiliations:** 1 Institute of Chemical Biology and Fundamental Medicine, Siberian Branch of Russian Academy of Sciences, Novosibirsk, Russia; 2 N.D. Zelinsky Institute of Organic Chemistry, Russian Academy of Sciences, Moscow, Russia; 3 Novosibirsk State University, Novosibirsk, Russia; 4 Aspergillus Unit, Institut Pasteur, Paris, France; 5 Molecular Mycology Unit, Institut Pasteur, Paris, France; National Cancer Institute at Frederick, UNITED STATES

## Abstract

β-(1→3)-D-Glucan is an essential component of the fungal cell wall. Mouse monoclonal antibodies (mAbs) against synthetic nona-β-(1→3)-D-glucoside conjugated with bovine serum albumin (BSA) were generated using hybridoma technology. The affinity constants of two selected mAbs, 3G11 and 5H5, measured by a surface plasmon resonance biosensor assay using biotinylated nona-β-(1→3)-D-glucan as the ligand, were approximately 11 nM and 1.9 nM, respectively. The glycoarray, which included a series of synthetic oligosaccharide derivatives representing β-glucans with different lengths of oligo-β-(1→3)-D-glucoside chains, demonstrated that linear tri-, penta- and nonaglucoside, as well as a β-(1→6)-branched octasaccharide, were recognized by mAb 5H5. By contrast, only linear oligo-β-(1→3)-D-glucoside chains that were not shorter than pentaglucosides (but not the branched octaglucoside) were ligands for mAb 3G11. Immunolabelling indicated that 3G11 and 5H5 interact with both yeasts and filamentous fungi, including species from *Aspergillus*, *Candida*, *Penicillium* genera and *Saccharomyces cerevisiae*, but not bacteria. Both mAbs could inhibit the germination of *Aspergillus fumigatus* conidia during the initial hours and demonstrated synergy with the antifungal fluconazole in killing *C*. *albicans in vitro*. In addition, mAbs 3G11 and 5H5 demonstrated protective activity in *in vivo* experiments, suggesting that these β-glucan-specific mAbs could be useful in combinatorial antifungal therapy.

## Introduction

The incidence of invasive fungal infections continues to increase, and successful treatment of the diseases remains a serious problem despite the development of more effective antifungal preparations with reduced toxicity [1,2]. Early detection of invasive fungal infections is extremely important for successful treatment. Invasive fungal infections in humans are caused mainly by the species from *Aspergillus*, *Candida*, *Cryptococcus*, and *Fusarium* genera. Structurally, fungal cells are protected by a cell wall composed of different polysaccharides; while establishing infection, this cell wall undergoes modification and rearrangement, during which some fragment of these polysaccharides are expected to be released [3]. One of the major and essential components of the fungal cell wall is β-(1→3)-D-glucan [4–6]. Detection and quantitative evaluation of this polysaccharide is an important challenge for clinical diagnosis, food control, and ecology monitoring. Currently, Glucatell and related kits for measurement of β-(1→3)-D-glucans with a glucan-reactive preparation of *Limulus* amebocyte lysate (LAL) [7–9] is widely used; however, it shows a high rate of false positive results for fungal infection [10]. Therefore, an antibody-based enzyme immune-assay (EIA) can be regarded as a practical alternative to the LAL-test in many cases, as it is less expensive and can be sufficiently sensitive to detect β-(1→3)-D-glucan in clinical samples [11]. Several EIAs were developed to date based on polyclonal and monoclonal antibodies [11–13] that were obtained against β-glucans and their BSA-conjugates. Their specificity was evaluated with the use of polysaccharide preparations isolated from natural sources, and therefore the tests were insufficiently characterized.

**Fig 1:**
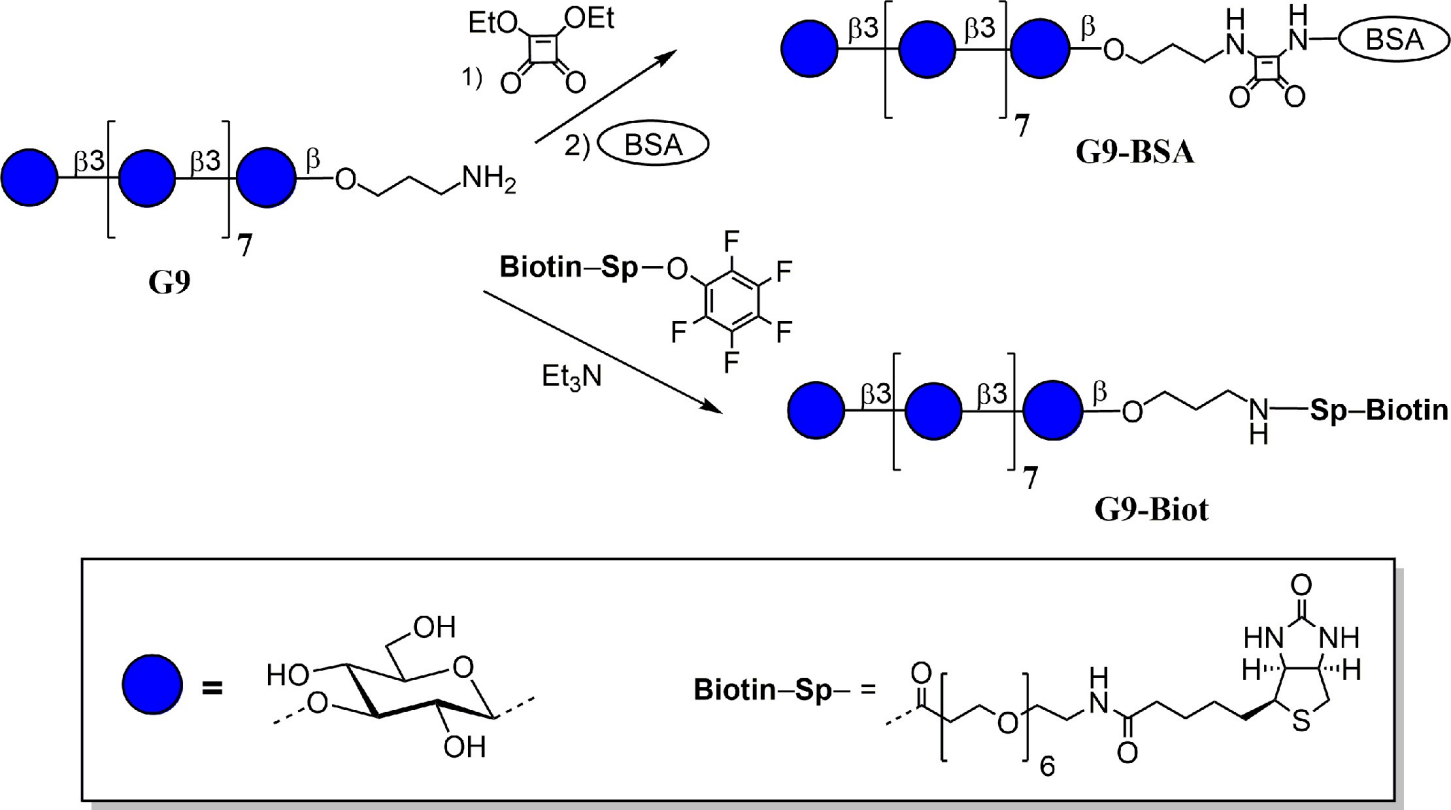
Structure of nonasaccharide G9 and its BSA (G9-BSA) and biotinylated (G9-Biot) conjugates used in mouse immunization and mAb screening; the carbohydrate sequences are represented according to symbol carbohydrate nomenclature [18].

**Fig 2:**
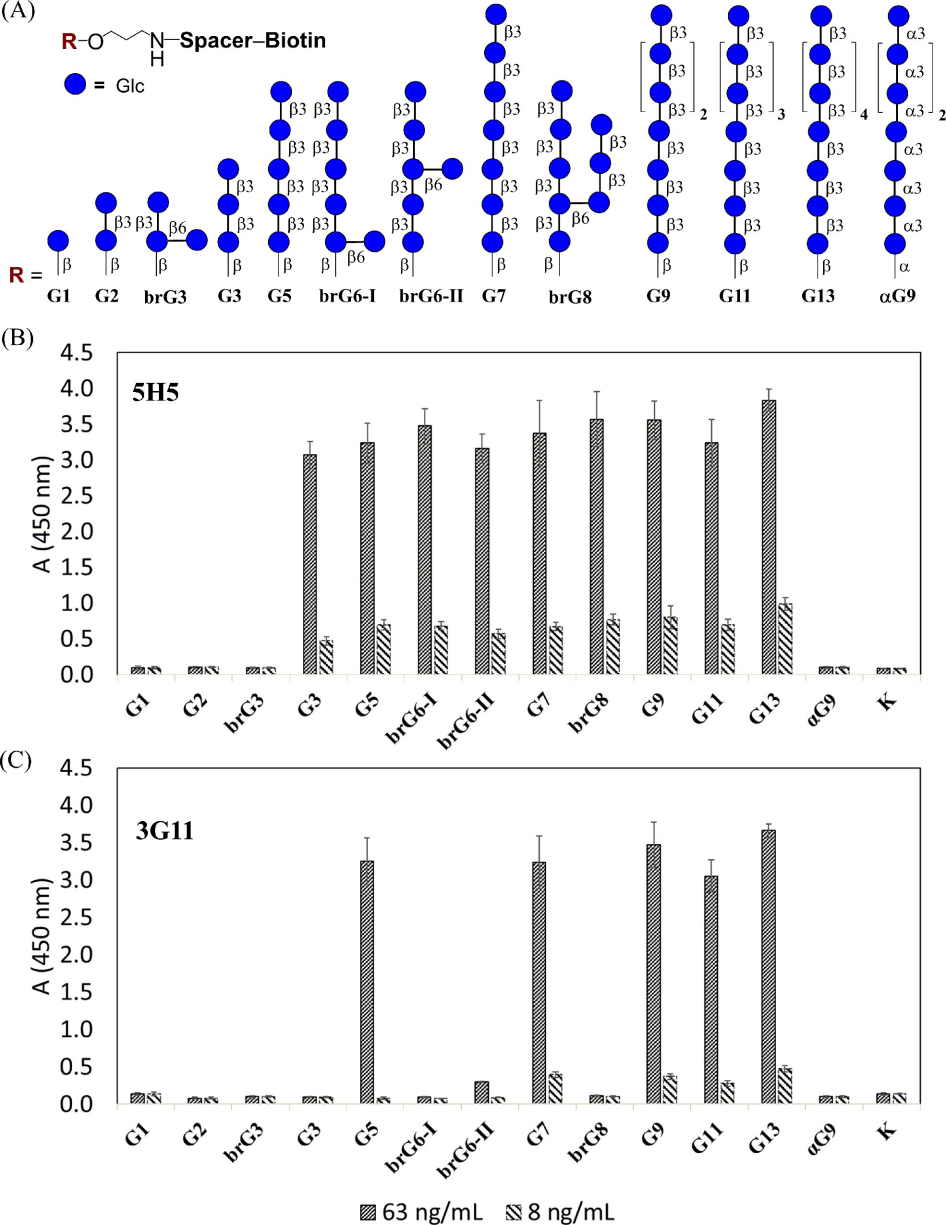
Investigation of oligosaccharide specificity of mAbs 3G11 and 5H5 using ELISA. (A) Composition of a thematic glycoarray built using linear (**G1**-**G13**) and branched (**brG3**, **brG6-I, brG6-II, brG8**) oligosaccharide ligands representing key structural elements of the β-(1→3)-D-glucan chain. The α-(1→3)-linked glucosaccharide **αG9** was used as a negative control. Assay for the carbohydrate specificity of 5H5 (B) and 3G11 (C) mAbs. All measurements were independently repeated twice in triplicate. The results are presented as the means ± s.d.

In this study, we describe selection and characterization of two anti-β-(1→3)-D-glucan monoclonal antibodies (5H5 and 3G11) that were developed with the use of nona-β-(1→3)-D-glucoside-BSA conjugate [14] **G9-BSA** (Fig 1). The nonaglucoside ligand in this preparation represents the linear fragments of β-(1→3)-D-glucan. The characterization of epitopes of mAbs 5H5 and 3G11 was performed for the first time with the use of a thematic glycoarray (Fig 2A) comprised of 13 biotinylated oligoglucoside ligands (from mono- to tridecasaccharide) representing key structural elements of linear and 3,6-branched β-(1→3)-D-glucans [15–17], which were fixed on the surface of a streptavidin-coated plate and used in an indirect ELISA. The 5H5 and 3G11 mAbs were generated with a goal to develop sandwich-like EIAs for detection of glucan in ecological, food, veterinary, and clinical samples. However, in this study, we showed the potential of these two mAbs for localizing β-(1→3)-D-glucan in the fungal cell wall, inhibiting fungal growth and in the combinatorial antifungal therapy.

## Materials and methods

### Biotinylated conjugates of synthetic oligosaccharides and Glc9-BSA immunogen

The synthesis of spacer-armed oligosaccharides related to β-(1→3)-D-glucan fragments has been described previously [15–17]. Bovine serum albumin (BSA) conjugate of nona-β-(1→3)-D-glucoside (**G9-BSA**) was prepared from parent aminopropyl glycoside (**G9**) using the squarate protocol [14] (Fig 1). According to MALDI TOF MS data, G9-BSA contained on average ~10 oligosaccharide chains per protein molecule.

Preparation of biotinylated conjugates from β-(1→3)-D-glucan ligands for the creation of glycoarrays (Fig 2A) was performed by treating parent aminopropyl glycosides with the active ester of biotin in dimethylformamide following the biotinylation protocol described previously [19]. Biotinylated glycoconjugates were isolated by gel-permeation chromatography on a Toyopearl HW-40(S) gel (Tosoh, Japan) column, eluted using 0.1 M acetic acid with 65–75% yields.

### Animals

Female BALB/c mice were purchased from the animal care facility in the Federal State Research Center of Virology and Biotechnology “Vector” (Koltsovo, Russia). Mice were housed with a normal light-dark cycle; food and water were provided *ad libitum*. All animal procedures were carried out in accordance with the recommendations for the protection of animals used for scientific purposes (EU Directive 2010/63/EU). Immunized mice were euthanized with an overdose of isoflurane (5%). These animal experiments were approved by the local Bioethics Committee of the Institute of Chemical Biology and Fundamental Medicine, Siberian Branch of Russian Academy of Sciences (ICBFM, SB RAS), Novosibirsk, Russia.

### Mouse immunization and mAbs selection

For immunization, 10 μg **G9-BSA** in 300 μL phosphate buffer saline (PBS), pH 7.4, emulsified with an equal volume of complete Freund’s adjuvant (Sigma-Aldrich, St. Louis, MO) were subcutaneously administered into 12–14-week-old female BALB/c mice (22–28 g). At two and four weeks, each mouse was additionally immunized intraperitoneally with 10 μg **G9-BSA** mixed with incomplete Freund’s adjuvant (Sigma-Aldrich, St. Louis, MO). Two weeks after the third immunization, mice were finally immunized with 10 μg of **G9-BSA** in 300 μL PBS, pH 7.4. After three days, antibody titer of the mice sera (obtained from orbital blood sample) against nona-β-(1→3)-D-glucoside conjugated with biotin (**G9-biot**) were checked by indirect ELISA. Those mice with the highest antibody titer were sacrificed and obtained splenocytes were fused with SP2/0 myeloma cells using PEG 2000 (Roche, Basel, Switzerland) according to the manufacturer’s protocol. The SP2/0 myeloma cell line was cultured in Iscove’s modified Dulbecco’s medium (Invitrogen, Waltham, MA) supplemented with 10% fetal bovine serum (Biolot, Russia) and antibiotics (0.1 mg/mL streptomycin and 100 IU/mL penicillin). Hybridomas were cultured and cloned in Iscove’s modified Dulbecco’s medium (Invitrogen, Waltham, MA) supplemented with 10% fetal bovine serum (Biolot, Russia), 5.7 μM azaserine (Sigma-Aldrich, St. Louis, MO), 100 μM hypoxanthine (Sigma-Aldrich, St. Louis, MO), 50 U/mL interleukin-6, and antibiotics (0.1 mg/mL streptomycin and 100 IU/mL penicillin). Hybridoma clones were selected by assaying the titer of mAbs in the supernatants by ELISA using **G9-biot**. Positive clones were additionally cloned two times by the limiting dilution method. Selected mAbs were propagated and purified using protein A chromatography (GE Healthcare, Chicago, IL), as described previously [20].

### The IgG subclass determination

To determine the IgG class of a mAb produced by the selected hybridoma clone, total RNA was isolated from the appropriate hybridoma cell line using RNeasy Mini kit (Qiagen, Venlo, Netherlands). A fragment of the gene encoding constant domain CH1 was amplified by RT-PCR using the primers 5′- CTTCCGGAATTCSARGTNMAGCTGSAGSAGTC-3′ [21] and 5′-GGGAAGTAGCCTTTGACAAGGC-3′ and sequenced in both directions.

### Purification and conjugation of mAbs

To obtain mAbs, 2×10^6^ hybridoma cells, producing anti-**G9** antibodies, were resuspended in 0.5 mL of sterile 0.9% NaCl and administered intraperitoneally into 20-week-old BALB/c mice. Selected mAbs 3G11 and 5H5 were purified by ammonium sulfate precipitation from ascitic fluids and then purified using protein A chromatography (GE Healthcare, IL). The purity and size of the purified IgG antibodies were examined by SDS-PAGE and Western blot analyses. Purified mAbs were resolved by 12.5% SDS-PAGE under reducing conditions and transferred onto a nitrocellulose membrane (Bio-Rad, CA). After blocking with 5% casein (skim milk powder) in PBS, the membrane was incubated with anti-mouse IgG alkaline phosphatase-conjugated goat IgG (Sigma Aldrich, MO). Immune complexes were visualized by a mixture of nitro blue tetrazolium (WVR, PA) and 5-bromo-4-chloro-3-indolyl-phosphate (Roche, Germany) for 20 min. The selected mAbs 3G11 and 5H5 were conjugated with horse-radish peroxidase (WVR, PA) using the optimized NaIO_4_ method as described previously [22].

### Oligosaccharide-specific indirect ELISA

For indirect ELISA, a 96-well Pierce streptavidin-coated plate was coated with 50 ng/well of **G9-biot** in 25 mM Tris-HCl, pH 7.5, with 150 mM NaCl, 0.05% Tween-20, and 0.3% BSA and incubated at 4°C overnight. Following, the wells were washed three times with wash buffer (25 mM Tris-HCl, pH 7.5 + 150 mM NaCl + 0.05% Tween 20 + 0.3% BSA), followed by adding mouse sera, hybridoma supernatants, or mAbs in appropriate dilutions and incubating at 37°C for 1 h. After washing three times with wash buffer, anti-mouse IgG alkaline phosphatase-conjugated goat IgG (Sigma Aldrich, USA) was added and incubated at 37°C for 1 h, followed by washing three times with the wash buffer and then AP-buffer (100 mM Tris-HCl, pH 9.5, with 100 mM NaCl and 5 mM MgCl_2_). The substrate 4-nitrophenyl phosphate was added, and the absorbance was measured at 405 nm using an iMark plate reader (Bio-Rad, USA).

### Glycoarray

The wells of 96-well Pierce streptavidin-coated plates were coated with 20 pmol/well of appropriate linear (**G1**-**G13**) and branched (**brG3**, **brG6-I, brG6-II, brG8**) biotin-tagged oligosaccharides (Fig 2) in 100 μL of wash buffer and then incubated for 2 h at 37°C as previously described [20,23,24]. After washing three times with wash buffer, the plates were incubated with mAbs 5H5 and 3G11 diluted in wash buffer (concentration 63, 8.0 ng/mL) for 1 h at 37°C. After washing three times with wash buffer, anti-mouse IgG rabbit IgG-horseradish peroxidase conjugate (Imtek, Russia) was added and incubated for 1 h at 37°C. After washing three times with wash buffer, the color was developed using TMB mono-component substrate (100 μL) for 15 minutes, and the reaction was stopped with 50 μL of 1 M sulfuric acid. The absorbance was measured at 450 nm using a MultiSkan GO plate reader (Thermo Fisher Scientific, USA). All measurements were independently repeated twice in triplicate. Results are presented as the means ± s.d.

### Sandwich anti-β-glucan ELISA

For sandwich ELISA, 10^8^ microbial cells were harvested by centrifugation, washed twice in 1 ml 0.9% NaCl, resuspended in 1 mL PBS, and sonicated as described previously [25] with our modifications. Sonications were done using a cell disintegrator Sonopuls Ultrasonic homogenizers HD 2070 (Bandelin, Germany) at 20 W and 20 kHz. Samples, of volumes 0.5 ml, were kept in an ice bath during cell disruption. Sonication treatments consisted of five periods of 20 sec followed by 1 min of resting to prevent overheating. In addition, supernatants of bacterial cultures were also assayed for their binding with mAbs 3G11 and 5H5 using a sandwich ELISA. The 96-well microtiter plates (Greiner, Austria) were coated with 200 ng/well mAb 3G11 or mAb 5H5 in PBS and incubated at 37°C for 1 h. After blocking with 5% casein (skimmed milk) in PBS, coated wells were washed three times with PBS containing 0.1% Tween-20 (PBST) and three times with PBS. Then, serially diluted (five-fold) opalescent microbial cell lysates or bacterial culture supernatants were added, and the plates were incubated at 37°C for 1 h. After subsequent washing three times with PBST and PBS, an indirect ELISA was performed with horseradish peroxidase-conjugated mAb 5H5 (100 μL). The assay was developed using 3,3',5,5'-Tetramethylbenzidine (TMB; WVR, PA) mono-component substrate (100 μL) for 15 minutes, and the reaction was stopped with 50 μL of 1 M sulfuric acid. The absorbance was measured at 450 nm using an iMark plate reader (BioRad, CA). ELISA was performed with two different preparations of microbial cell lysates, and each time in triplicate. The results are presented as the means ± s.d.

### Affinity constant measurement

The affinity of mAbs to oligosaccharide antigens was determined by a Surface Plasmon Resonance-based biosensor assay (SPR) using a ProteOn XPR36 system (Bio-Rad, USA). PBS with 0.005% Tween-20 (PBSTmin) was used as a system running buffer. Vertical channels L3 and L4 of a GLC sensor chip were coated covalently with streptavidin at the 90−110 response unit (RU) level. Biotinylated oligosaccharides diluted to 3 μg/mL in PBSTmin were immobilized onto streptavidin-coated channel L3 for 50 seconds resulting in a 5 RU level, while L4 was used as a reference channel. To perform affinity measurements, three-fold dilutions of mAbs were analyzed at a flow rate of 25 μL/min. The duration of both the association and dissociation was 300 seconds. Antibody concentration ranges were determined based on data from scouting experiments, in which the starting concentration of mAbs was 180 nM. All experiments were repeated three times. The chip surface was regenerated with 100 mM citric acid. Global analysis of experimental data based on a single-site model was performed using the ProteOn Manager v. 3.1.0 software. Affinity constants were calculated as K_D_ = k_d_/k_a_.

### Microorganisms

Fungal strains *Candida albicans* ATCC 10231 and bacterial strains *Enterococcus faecalis* ATCC 51299, *Escherichia coli* ATCC 25922, *Proteus mirabilis* ATCC 25933, *Pseudomonas aeruginosa* ATCC 27853, *Salmonella enterica* ATCC 14028, and *Staphylococcus aureus* ATCC 25923 were purchased from ATCC and maintained in the Collection of Extremophilic Microorganisms and Type Cultures (CEMTC) of ICBFM, SB RAS. Other microorganisms, including *Aspergillus fumigatus*, *Candida parapsilosis*, *Candida tropicalis*, *Candida dubliniensis*, *Debaryomyces hansenii*, *Penecillium polonicum*, *Penecillium solitum*, *Alcaligenes faecalis*, *Bifidobacterium infantis*, *Bifidobacterium longum*, and *Lactobacillus plantarum* were isolated from clinical samples or natural habitats and characterized by 16S rRNA gene sequencing and biochemical properties using a biochemical analyzer (GEN III OmniLog Combo Plus System, Biolog, USA) in CEMTC. Fungi, including *A*. *fumigatus*, *C*. *albicans*, *C*. *parapsilosis*, *C*. *tropicalis C*. *dubliniensis*, *D*. *hansenii*, *P*. *polonicum*, and *P*. *solitum*, were grown in Sabouraud Dextrose broth (SDB) at room temperature overnight (*Candida* spp.) or for 44 hrs (all other fungi). Several bacterial species, including *B*. *infantis*, *B*. *longum*, and *L*. *plantarum*, were cultivated in Blaurock medium, while other bacteria, namely *A*. *faecalis*, *E*. *faecalis*, *E*. *coli*, *P*. *mirabilis*, *P*. *aeruginosa*, *S*. *enterica*, and *S*. *aureus*, were propagated in Luria-Bertani broth at 37°C. All these bacteria were grown in a shaken incubator for overnight and then used in experiments.

### Immunolabeling and confocal microscopy

For immunolabeling, fungal and bacterial cells were allowed to adhere on slides and were then fixed with 2.5% *para*-formaldehyde overnight at 4°C. Slides with fixed cells were washed with PBS twice and blocked with 3% BSA in PBS for 1 h. Then, cells were washed and incubated with 5 μg/mL mAb diluted in PBS with 3% BSA for 1 h at 37°C. After washing, cells were stained with Alexa Fluor-488- or Cy5-conjugated chicken anti-mouse IgG (H+L) antibodies (Life Technologies) or FITC-conjugated anti-mouse IgG (Sigma) diluted 1:500 in PBS with 3% BSA. Samples were mounted using Prolong Diamond Antifade and examined under a Carl Zeiss LSM 710 laser scanning microscope (Carl Zeiss, Germany). Observations were performed using oil 63× objectives, and images were captured at 488 nm in green or 670 nm in red and differential interference contrast (DIC) channels. ZEN black edition software (Carl Zeiss, Germany) was used in the confocal microscope to visualize images.

### Germination inhibition assay

3G11 or 5H5 (5 μl; stock solution concentration, 1 mg/mL) was added to 5 × 10^3^ conidia in 5 μl tween-water (0.05%), and the mixture was placed on Sabouraud agar medium spread over a glass-slide. The slides were incubated at 37°C and after 6 h, they were observed each hour for germination under the microscope. On each slide three inoculum mixtures were spotted, and at least four different positions were imaged wherein germinated and non-germinated conidia were counted. For control samples, 5 × 10^3^ conidia in 5 μl tween-water mixed with 5 μl PBS was plated on Sabouraud agar medium, and the germination was recorded from 6 h onwards. This assay was performed in triplicate, and at least hundred conidia were counted each time.

### Phagocytotic assay

A phagocytotic assay was performed for *A*. *fumigatus* conidia using human monocyte derived macrophages (HMDM) obtained as described previously [26,27]. *A*. *fumigatus* conidia were harvested from 12-15-day-old malt-agar slants using 0.05% aqueous Tween-20 and washed twice with aqueous Tween-20. Swollen conidia were obtained by inoculating 1 × 10^8^ conidia in 50 mL of Sabouraud liquid medium. The conidia were incubated at 37°C in a shaken incubator (150 rpm) for 5 h, followed by harvesting of the conidia by centrifugation and washing twice with water. To label the conidia with fluorescein isothiocyanate (FITC), 1 mg/mL FITC was diluted 1:10 with carbonate buffer of pH 9.6, and 200 μl diluted FITC was then added to 1 × 10^8^ swollen conidia. The conidia were incubated at ambient temperature for 30 min, and the supernatant was discarded after centrifugation. The pellet was washed thoroughly with carbonate buffer to remove excess of FITC and then suspended in PBS. 3G11 (5 μl; stock solution concentration, 1 mg/mL) was then added to 1x10^6^ FITC-labelled swollen conidia (wherein there is exposure of the β-(1→3)-D-glucan on the surface). The mixture was incubated for 30 min and then suspended in incomplete RPMI. These conidia were added to HMDM (obtained upon seeding 2x10^6^/well peripheral blood mononuclear cells isolated from healthy donors) adhered to culture plates and incubated at 37°C in a CO_2_ incubator for 1 h. Following, the culture supernatant was discarded, the wells were washed twice with incomplete RPMI, fixed with 2.5 μ% *para*-formaldehyde for 10 min, and washed with incomplete RPMI. Calcofluor white solution (10 μg/mL in PBS) were added to label conidia outside macrophages [26], washed with PBS, and viewed under fluorescent microscope. 3G11 untreated conidia were used as the control. The assay was performed with HMDM obtained from three different donors; for each sample, there were duplicate wells on the culture plates, and from both wells, images were taken to count at least one hundred swollen conidia.

### Antifungal assays

Approximately 10^5^ colony forming units/mL (CFU/mL) from the overnight *C*. *albicans* culture were inoculated into fresh Sabouraud dextrose broth. Fluconazole, mAb 3G11, and mAb 5H5 were diluted in 0.9% NaCl, and two-fold dilutions of mAb 3G11, mAb 5H5, or fluconazole were added to the *C*. *albicans* culture. The mixtures were incubated at room temperature with shaking for 18 h. Then, aliquots were withdrawn, diluted in SDB, and serial dilutions of the mixtures were plated onto Sabouraud Dextrose agar. The plates were incubated overnight at room temperature, and the number of colonies that grew on the plates was counted. When fluconazole and a mAb were added simultaneously, fluconazole was used at a concentration of 50 μg/mL, while two-fold dilutions of the mAb were used. In this experiment, fluconazole at a concentration of 100 μg/mL and sterile 0.9% NaCl were used as positive and negative controls, respectively. All experiments were carried out twice in triplicate. The limit of quantification by this method was 100 CFU/mL.

### *In vivo* protection in mice

Protection of mAbs 3G11 and 5H5 was studied in a mouse model of systemic candidiasis, described previously [28,29] with modifications. Briefly, *C*. *albicans* ATCC 10231 cells, grown in SBD at 28°C overnight, were harvested by centrifugation, washed with sterile 0.9% NaCl, and counted in a hemocytometer. Six week-old female BALB/c mice were administered intraperitoneally 0.5 mL of mAbs 3G11 or 5H5 at a dose of 150 μg/mouse; control mice received an irrelevant anti-tick-borne encephalitis mouse mAb (IgG1) [30] at the same dose or 0.5 mL 0.9% NaCl. Two hours later, all mice were infected intravenously with 0.2 mL of *C*. *albicans* suspension in 0.9% NaCl (5 × 10^6^ cells per mouse). Each experimental and control group included eight mice. The mice were observed for 60 days after infection to assess the survival rate. Data were analyzed using the on-line service available at https://www.evanmiller.org/ab-testing/survival-curves.html and https://www.graphpad.com/quickcalcs.

### Statistics

All measurements for oligosaccharide-specific indirect ELISA and glycoarray were independently repeated twice in triplicate. The data are presented as the mean ± standard deviation (s.d.). Statistical analysis of germination inhibition assay and phagocytotic assay was performed by one-way ANOVA; *, p<0,05 and **, p<0,005. The differences in CFU counts in antifungal *in vitro* assays were computed using a two-tailed Student’s t- test; p < 0.001 was regarded as significant. The Fisher’s exact test was used to compare the survival rates between the differently treated animal groups, whereas differences in the survival times were compared using the log-rank test.

## Results and discussion

### Anti-β-(1→3)-D-glucan mAbs generation and characterization

To generate mAbs specifically recognizing fungal β-(1→3)-D-glucan, the synthetic β-(1→3)-linked nonaglucoside **G9** was selected as target ligand. The conjugates of this oligosaccharide demonstrated high immunogenicity [14]. Moreover, theoretical and experimental conformational studies showed that the nonasaccharide sequence is long enough to mimic natural β-(1→3)-linked glucan [31]. Using synthetic nonasaccharide **G9**, immunogen **G9-BSA** was prepared by conjugation of corresponding aminopropyl glycoside with BSA using the squarate protocol (Fig 1). The biotin-tagged nonasaccharide **G9-Biot** required for selection of mAbs was prepared by treating amine with activated ester [19].

To select hybridomas producing mAbs against β-(1→3)-D-glucan, BALB/c mice were immunized four times with synthetic **G9-BSA**. Four, eight, and twelve weeks after the first immunization, mouse sera were screened by indirect ELISA to assess the level of anti-β-(1→3)-D-glucan antibodies. To prevent selection of anti-BSA mAbs, **G9-biot** was used as an antigen in the assay. Final titers of anti-β-(1→3)-D-glucan IgG antibodies ranged from 1:40000 to 1:200000 in the sera of different mice. Individual hybrid clones (n = 634) were screened for their binding with **G9-biot**, and ten hybridomas secreting specific mAbs were selected.

The IgG class of selected mAbs was determined as described previously [20] based on the sequences of PCR fragments encoding the constant domain CH1. Nine of the selected mAbs belonged to the IgG1 class, while mAb 5H5 was from the IgG3 class. Light chains of all the selected mAbs belonged to the kappa family.

**Fig 3:**
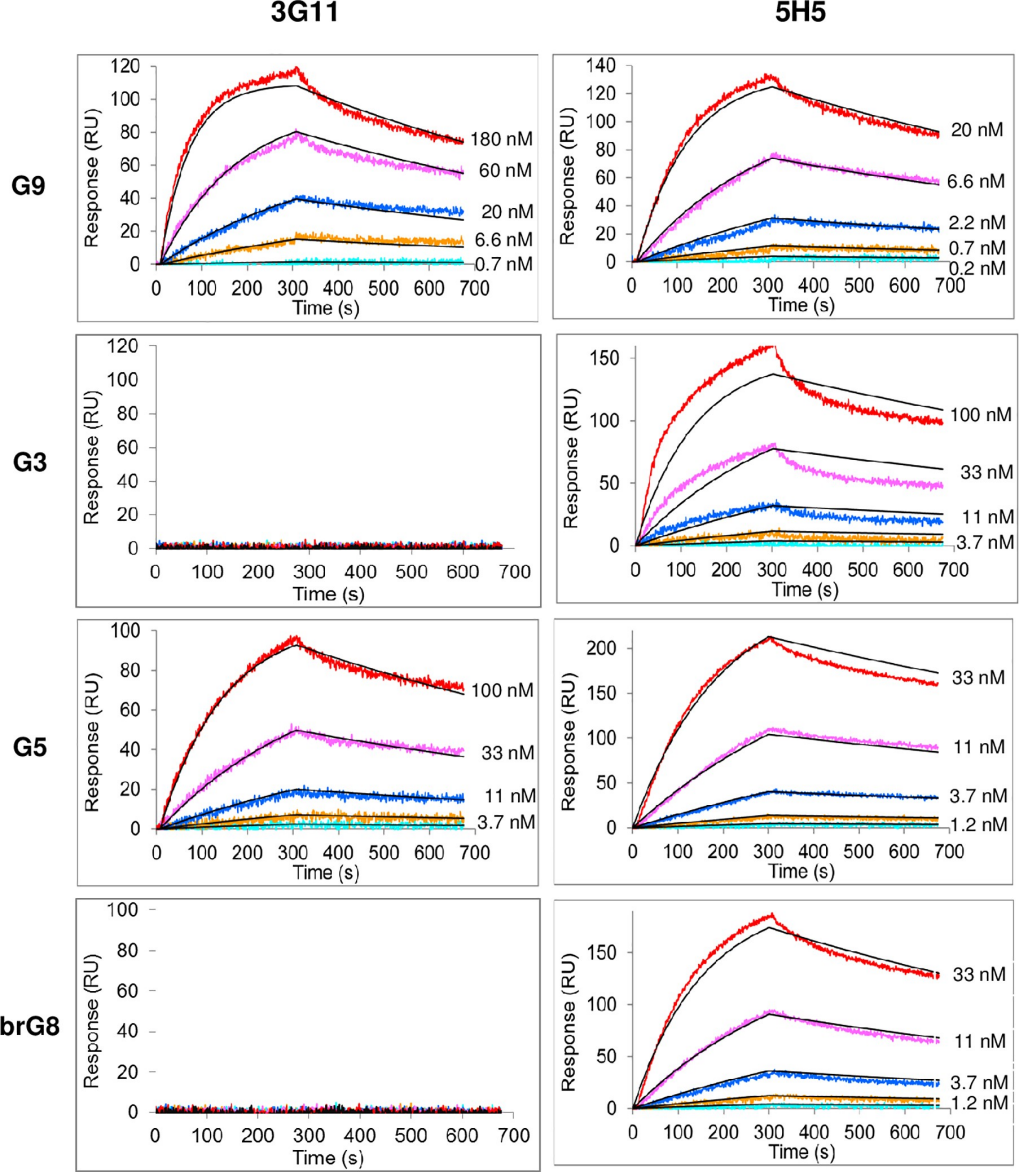
Binding of mAbs 3G11and 5H5 with biotinylated oligosaccharide ligands assayed by SPR. Serial three-fold dilutions of mAb 3G11 (left side) and mAb 5H5 (right side) were used as analytes. Experimental data are shown as color lines; fitted data are depicted as black curves; fitted traces are depicted as smooth black lines. Starting antibody concentrations were determined in scouting experiments.

Affinity of the purified selected mAbs was measured in a label-free SPR biosensor assay using **G9-biot** as the ligand. Two mAbs, 3G11 and 5H5, demonstrated the highest binding characteristics, while the affinity constants of the other selected mAbs were > 10^−6^ M. A global analysis of the interaction between mAbs 3G11 and 5H5 and the ligand demonstrated a good quality fit (Fig 3), and affinity constants were calculated as K_D_ = (1.1 ± 0.1) × 10^−8^ M for mAb 3G11 and K_D_ = (1.9 ± 0.2) × 10^−9^ M for mAb 5H5. The kinetic parameters and affinity constants for binding with biotinylated **G9-biot** are indicated in Table 1. As affinity constants of other selected mAbs were not acceptable, they were not used in further experiments.

**Table 1:** The kinetic parameters and affinity constants of mAbs 3G11 and 5H5 for binding with linear tri- (G3), penta- (G5) and nonaglucoside (G9) and branched octaglucoside brG8.

mAb	Synthetic glucosides			
	G3	G5	brG8	G9
**3G11**	K_D_ > 1 mM	K_D_ = 12 ± 1 nM	K_D_ > 1 mM	K_D_ = 11 ± 1 nM
		k_a_ = 6.4 × 10^4^ M^-1^s^-1^		k_a_ = 8.2 × 10^4^ M^-1^s^-1^
		k_d_ = 8.5 × 10^−4^ s^-1^		k_d_ = 8.8 × 10^−4^ s^-1^
**5H5**	K_D_ = 9.0 ± 3.0 nM	K_D_ = 4.0 ± 0.5 nM	K_D_ = 4.5 ± 0.5 nM	K_D_ = 1.9 ± 0.2 nM
	k_a_ = 7.2 × 10^4^ M^-1^s^-1^	k_a_ = 1.4 × 10^5^ M^-1^s^-1^	k_a_ = 1.7 × 10^5^ M^-1^s^-1^	k_a_ = 4.2 × 10^5^ M^-1^s^-1^
	k_d_ = 6.3 × 10^−4^ s^-1^	k_d_ = 5.6 × 10^−4^ s^-1^	k_d_ = 7.8 × 10^−4^ s^-1^	k_d_ = 8.1 × 10^−4^ s^-1^

### Epitope specificity of mAbs 3G11 and 5H5

The carbohydrate specificity of mAbs 3G11 and 5H5 was investigated using a library of thirteen synthetic oligosaccharides (see Fig 2B and 2C) representing distinct linear (**G1**-**G13**) and branched (**brG3**, **brG6-I, brG6-II, brG8**) structural fragments of β-(1→3)-D-glucan. These ligands as biotinylated conjugates were immobilized on the surface of the streptavidin-coated plate, and mAbs were assayed on the formed glycoarray at concentrations of 8 and 63 ng/mL.

The antibodies demonstrated different carbohydrate specificity profiles. 5H5 was less selective and recognized all tested ligands except mono- (**G1**), diglucoside (**G2**) and branched triglucoside (**brG3**). 3G11 recognized all linear oligoglucosides longer than a pentamer (**G5**, **G7**, **G9**, **G11**), but it did not recognize branched oligoglucosides **brG6-I, brG6-II, brG8**, showing differences between the affinities of both mAbs. The mAbs did not recognize α-(1→3)-linked nonasaccharide ligand α**G9**. These results indicated that mAb 5H5 minimally recognizes the β-(1→3)-linked trisaccharide moiety, while 3G11 required the presence of a linear pentasaccharide fragment for effective binding.

These results were confirmed by SPR assays (**Fig 3**, Table 1). 5H5 recognized all tested linear antigens, though with different affinities, and the binding effectiveness increased (9 nM → 4 nM → 1,9 nM) with increasing length of the oligosaccharide **G3** → **G5** → **G9** (Table 1). By contrast, 3G11 specifically bound to penta-**G5** and nonaglucoside **G9** with almost equal affinities (Table 1). Probably, 5H5 is able to recognize a smaller fragment of β-(1→3)-D-glucan that leads to less specificity of this mAb compared to 3G11.

### Immunofluorescence microscopy

**Fig 4:**
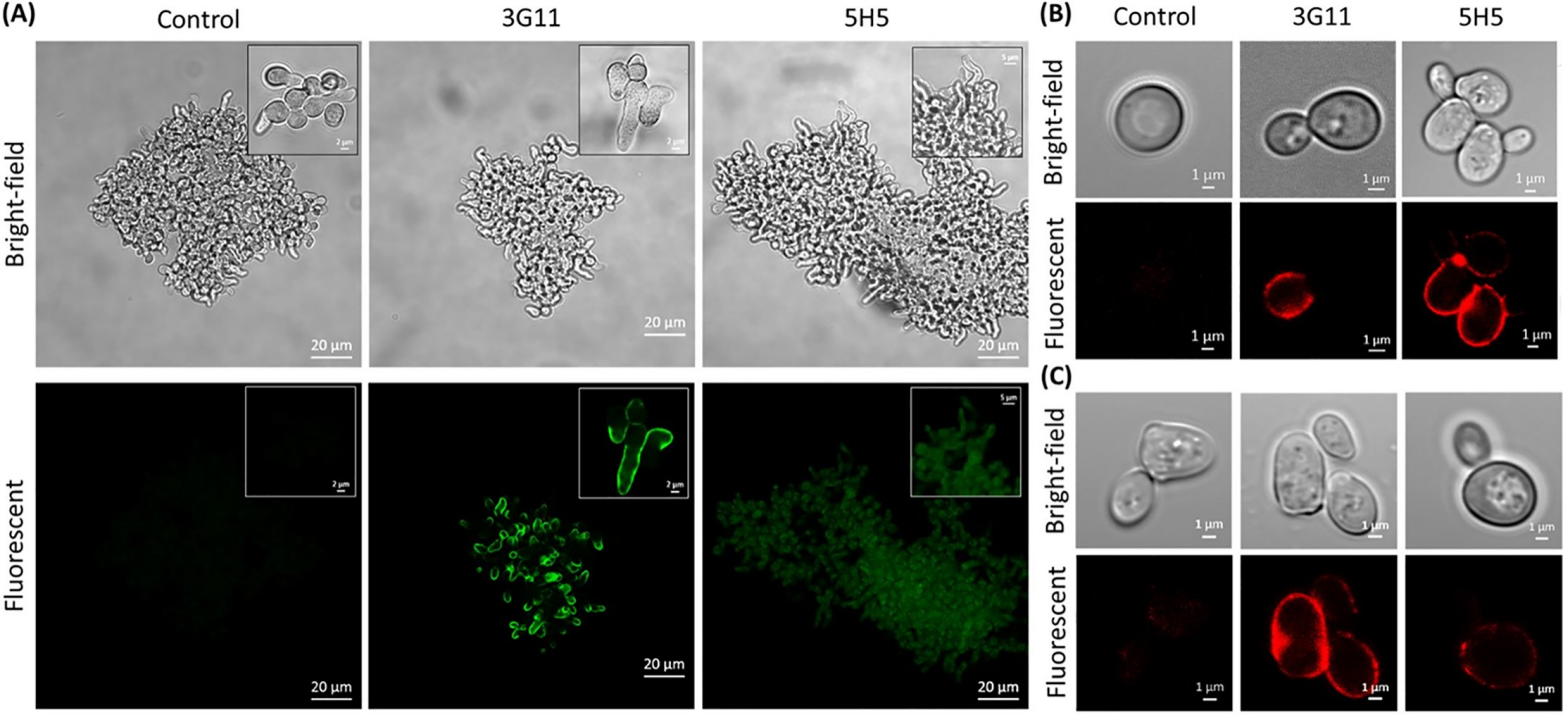
Immunofluorescence microscopy of different fungal species: immunolabelling for *A*. *fumigatus* (A), *C*. *albicans* (B) and *S*. *cerevisiae* (C) by mAbs 3G11 and 5H5.

The ability of mAbs 3G11 and 5H5 selected against synthetic β-glucan oligosaccharide to specifically bind with the fungal cell wall was demonstrated by immunofluorescence confocal microscopy. Both mAbs 3G11 and 5H5 recognized *Candida* species: *C*. *albicans*, *C*. *parapsilosis*, *C*. *tropicalis C*. *dubliniensis*, including medically important ones, as well as *A*. *fumigatus*, *P*. *polonicum*, *P*. *solitum*, *D*. *hansenii*, and *S*. *cerevisiae* (see Fig 4 and S1 File). Confocal images of *A*. *fumigatus*, *C*. *albicans* and *S*. *cerevisiae* are presented in Fig 4. As can be seen, there was weak binding of 5H5 with *A*. *fumigatus* germinating conidia, while 3G11 could bind to only the budding cell wall of *C*. *albicans*. 5H5 could bind the mother cell wall, and both 3G11 and 5H5 could label *S*. *cerevisiae*, although labeling with 5H5 was weaker. This clearly indicates differential distribution of the ligands of 3G11 and 5H5 on these fungal cell walls. Notably, the microbe-mAb interaction time was the same in experiments with all tested microorganisms.

In addition to fungal cell walls, β-glucans are also a natural component of the bacterial cell wall. To evaluate the specificity of mAbs 3G11 and 5H5, their binding with several gram-positive (*B*. *infantis*, *B*. *longum*, *E*. *faecalis*, *L*. *plantarum*, and *S*. *aureus*) and gram-negative (*A*. *faecalis*, *E*. *coli*, *P*. *mirabilis*, *P*. *aeruginosa*, and *S*. *enterica*) bacterial cells was examined by immunofluorescence confocal microscopy (S1 File). The images demonstrated that both mAbs 3G11 and 5H5 did not label all the tested bacteria. Importantly, mAbs 3G11 and 5H5 did not recognize *A*. *faecalis* and *P*. *aeruginosa*, which are known to produce β-(1→3)-D-glucan that causes a false positive detection of invasive fungal infections with the Fungitell assay [32]. The lack of recognition by mAbs 3G11 and 5H5 in the case of the bacteria could be attributed to the fact that bacterial β-(1→3)-D-glucans are mostly linear (with small amounts of 2,3-branchings) or cyclic [33], while those of fungi are partially 3,6-branched [34]. Conformational differences between bacterial cyclic or linear β-(1→3)-D-glucans (the latter may form triple helical fibrillar structures [31]) and fungal glucans can cause the observed discrimination in the recognition by the studied mAbs.

### Sandwich anti-β-glucan ELISA

**Fig 5:**
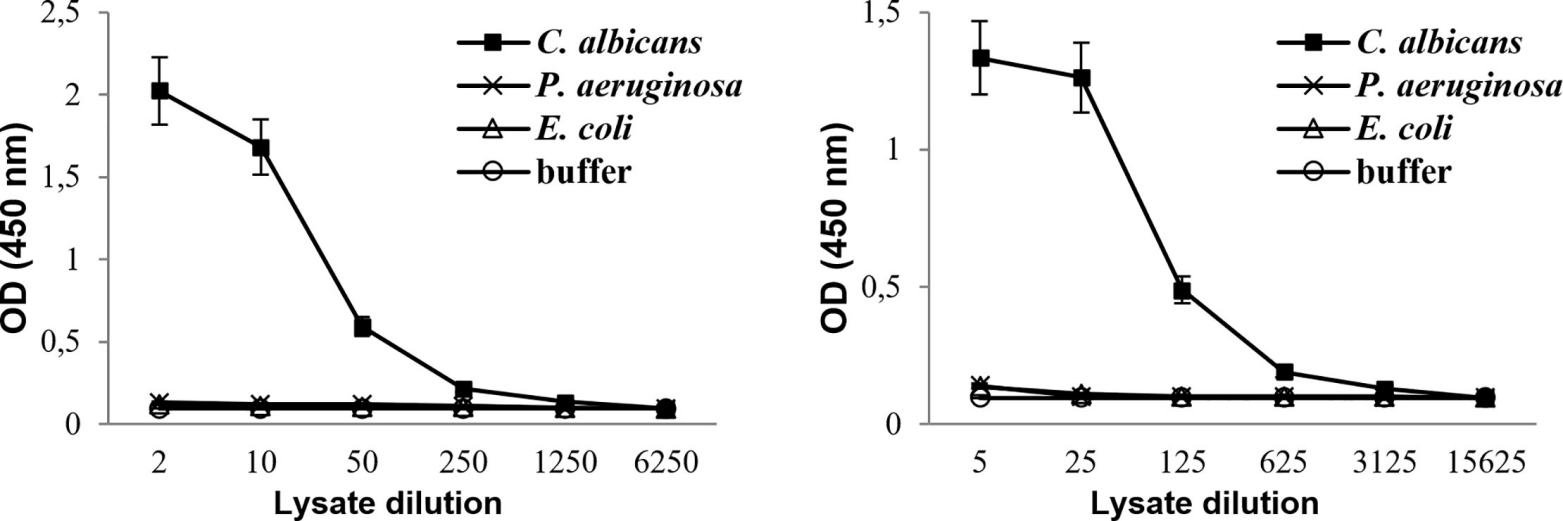
Binding of fungal and bacterial cell lysates with mAbs 3G11 and 5H5. Sandwich enzyme-linked immunosorbent assay (ELISA) with anti-**G9** antibodies: the wells of microtiter plates were coated with 200 ng mAb 3G11 (**A**) or mAb 5H5 (**B**) and incubated with serially diluted sonicated suspension of indicated microbial cultures; ELISA was performed with horseradish peroxidase-conjugated mAb 5H5.

To confirm specific binding of mAbs 3G11 and 5H5 with *C*. *albicans*, a sandwich ELISA was performed. Sonicated suspensions of *C*. *albicans*, *E*. *coli*, or *P*. *aeruginosa* were serially diluted, added to mAb 3G11 or mAb 5H5 coated on ELISA plate wells, and developed using mAb 5H5 HRP-conjugate. The results showed that both mAbs did not bind *E*. *coli* and *P*. *aeruginosa* cell lysates, but they recognized sonicated suspension of *C*. *albicans* (Fig 5).

Since bacterial β-(1→3)-glucans were shown to be mainly secretory components of bacterial cells [35] and could be accumulated in growth medium, bacterial culture supernatants were additionally assayed in sandwich ELISA (S1 Fig). The data indicated that *E*. *coli* and *P*. *aeruginosa* culture supernatants were not revealed by both mAb 3G11 and mAb 5H5 and confirmed results was demonstrated by immunofluorescence confocal microscopy.

### Antifungal assay

**Fig 6:**
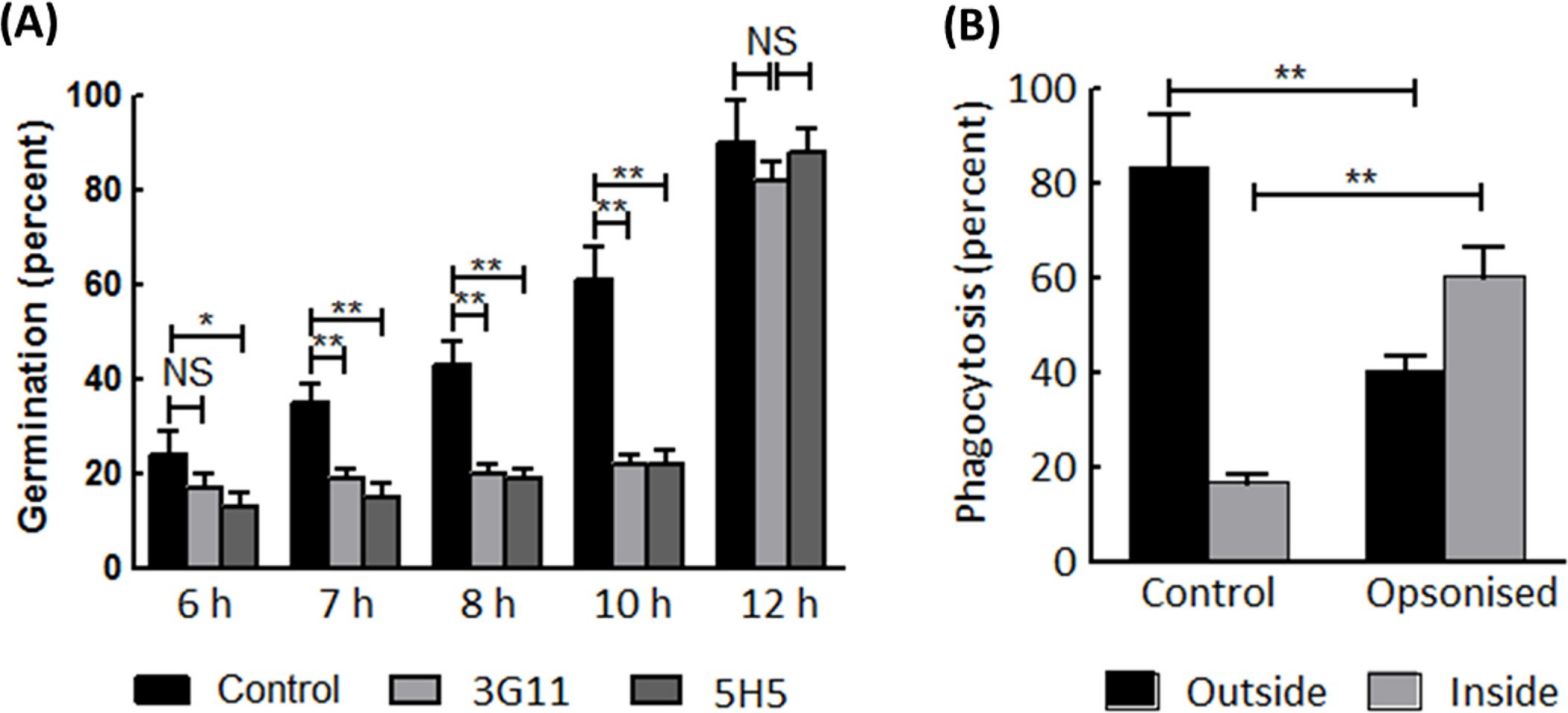
Antifungal activity of mAbs 3G11 and 5H5. **(A)** *A*. *fumigatus* germination inhibition assay. Conidia were treated with 3G11 or 5H5 (PBS buffer was used as a control) and placed on Sabouraud agar medium spread on a glass slide. Conidial germination was monitored after 6 h of incubation at 37°C. The incubation time is shown on the X-axis. The assay was repeated three times, and at least hundred conidia were counted each time. **(B)** Phagocytotic assay. Swollen conidia (FITC-labelled) were treated with 3G11 for 60 min followed by feeding to human monocyte derived macrophages (HMDM). After a further 60 min of incubation at 37°C in a CO_2_ incubator, non-phagocytosed conidia were differentiated from phagocytosed FITC-labelled conidia by calcofluor white staining. Images were taken from different places to count at least one hundred swollen conidia in each experiment. Statistical analysis was performed by one-way ANOVA; *, p<0,05 and **, p<0,005.

A β-(1→3)-D-glucan mAb with antifungal activity has been previously reported [28,36]. To assess possible antifungal activity, both 3G11 and 5H5 mAbs were tested *in vitro* for the inhibition of *A*. *fumigatus*. As shown in the Fig 6A, both 3G11 and 5H5 showed growth inhibition during the initial hours of growth, from six hours up to ten hours. We then checked the capacity of these antibodies to facility phagocytosis of *A*. *fumigatus* conidia by human monocyte derived macrophages. As 5H5 did not show efficient binding with *A*. *fumigatus* germinating conidia, we used only 3G11 for this study. Indeed, pretreatment of *A*. *fumigatus* conidia with 3G11 accelerated conidial phagocytosis by HMDM during the one hour of conidia-macrophage interaction (Fig 6B).

**Fig 7:**
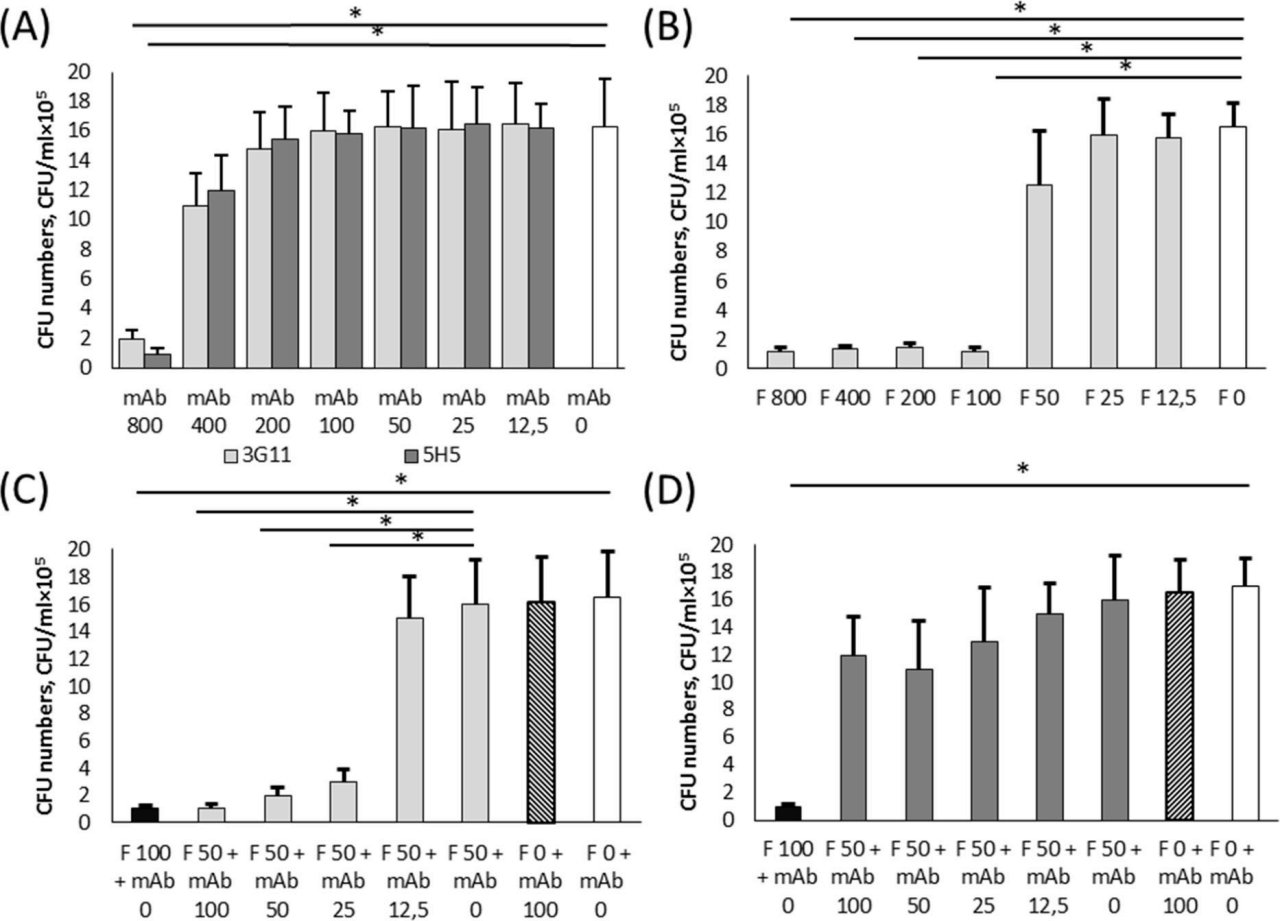
Antifungal activity of mAbs 3G11 and 5H5 and fluconazole. Two-fold dilutions of mAbs 3G11 or 5H5 **(A)**, fluconazole **(B)**, fluconazole with mAb 3G11 **(C)**, or fluconazole with mAb 5H5 **(D)** were added to an overnight *C*. *albicans* culture (10^5^ CFU/mL), and the mixtures were incubated at room temperature with shaking for 18 h. After incubation, aliquots were collected, serially diluted and plated on Sabouraud Dextrose agar, and the plates were incubated overnight at room temperature. The colonies, which grew on the plates, were counted. All experiments were carried out twice in triplicate. When fluconazole and a mAb were added simultaneously, fluconazole at a concentration of 100 μg/mL and sterile 0.9% NaCl were used as positive and negative controls, respectively. The limit of quantification by this method was 100 CFU/mL. The data are indicated as a mean value ± s.d. A two-tailed Student’s t- test was used to determine significance; *p < 0.01. Abbreviations: F.–Fluconazole; mAb–appropriate mAb; numbers indicate fluconazole and mAbs concentrations in μg/mL.

In addition, the anti-fungal activity of mAbs 3G11 and 5H5 was tested *in vitro* for the growth inhibition of *C*. *albicans*. An overnight *C*. *albicans* culture was adjusted in SDB to contain 10^5^ CFU/mL, mixed with two-fold serial dilutions of mAbs 3G11 or 5H5, starting at 800 μg/mL, and cultivated for 18 h. (Fig 7A). After cultivation, aliquots were titrated and plated on Sabouraud Dextrose agar plates, and colonies were counted after overnight incubation. The results indicated that both mAbs inhibited *C*. *albicans* growth when they were added only at a concentration of 800 μg/mL. The addition of both mAbs at a concentration of 400 μg/mL insignificantly decreased *C*. *albicans* cell growth, and the other concentrations were totally ineffective (Fig 7A). Next, the inhibition concentration of the well-known commercial antifungal preparation fluconazole was assessed in similar experiments (Fig 7B). *C*. *albicans* (10^5^ CFU/mL) was grown overnight with two-fold serial dilutions of fluconazole starting at 800 μg/mL. The addition of fluconazole at concentrations of 100–800 μg/mL led to inhibition of *C*. *albicans* growth, whereas concentration of 50 μg/mL only insignificantly decreased cell growth (Fig 7B). Finally, the possibility of cooperative growth inhibitory activity between fluconazole and mAb 3G11 or mAb 5H5 was examined. In these experiments, 50 μg/mL of fluconazole, which was sub-inhibitory concentration, was mixed with two-fold serial dilutions of mAbs 3G11 or 5H5, starting at 100 μg/mL, and these mixtures were added to a fresh overnight *C*. *albicans* cultures. Fluconazole at a concentration of 100 μg/mL and 0.9% NaCl were used as the positive and negative controls. There results indicated that mAb 3G11 provided cooperative *C*. *albicans* growth inhibitory action with fluconazole at mAbs 3G11 concentrations of 25–100 μg/mL (Fig 7C). Unlike mAb 3G11 with fluconazole, mAb 5H5 showed a weak co-operative *C*. *albicans* growth inhibitory activity (Fig 7D).

### Protective efficacy of mAbs 3G11 and 5H5

**Fig 8:**
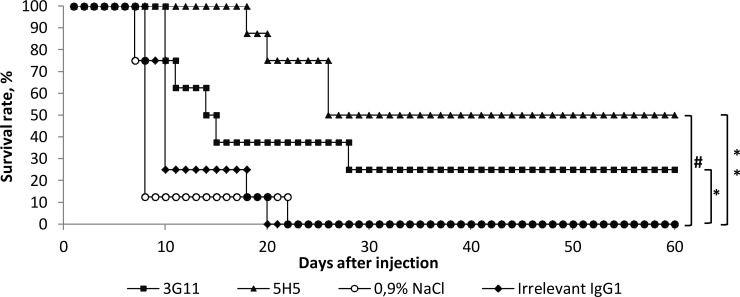
Protection of mice by mAbs 3G11 and 5H5 against fungal infection. BALB/c mice were administered (i.p.) once the indicated mAbs (150 μg/mouse in 0.5 mL 0.9% NaCl) or 0.5 mL 0.9% NaCl, and 2 h later, the mice received a lethal dose of *C*. *albicans* (5 × 10^6^ CFU/mouse). The survival rate of mice treated with mAb 5H5 was significantly higher than that of mice injected with 0.9% NaCl or an irrelevant mAb; #p < 0.05. The survival time of mice passively immunized with mAb 3G11 or mAb 5H5 was significantly higher than that of mice administered with 0.9% NaCl or an irrelevant mAb; *p < 0.01, **p < 0.001.

To test the protective efficacy of mAbs 3G11 and 5H5, a mouse model of systemic candidiasis [28,29] was used. Four groups of female BALB/c mice received a single pre-exposure injection of mAb 3G11, mAb 5H5, irrelevant mAb (IgG1), or 0.9% NaCl and then were infected with a lethal dose of *C*. *albicans*. As expected, no protection was observed when mice were administered with irrelevant mAb and 0.9% NaCl (Fig 8). The survival rate of mice treated with mAb 5H5 was significantly higher than the survival rates of animals, received irrelevant mAb or 0.9% NaCl. Moreover, a single injection of mAbs 3G11 and 5H5 resulted in a significant increase in the survival times, compared to those in mice injected with irrelevant mAb or 0.9% NaCl (Fig 8). The higher protective efficacy of mAb 5H5 compared to mAb 3G11 can be explained by its higher affinity (Table 1) and broader antigenic specificity, as, in contrast to mAb 3G11, mAb 5H5 effectively bound to all linear β-(1→3)-linked oligoglucosides longer than a trimer and recognized branched oligoglucosides **brG6-I**, **brG6-II**, **brG8** (Fig 2). In addition, mAb 5H5 belongs to the IgG3 family, which is known to induce antibody-dependent cellular cytotoxicity (ADCC) and antibody-dependent cellular phagocytosis (ADCP) more effectively than IgG1 [37]. In *C*. *albicans* infection model 5H5 showed better protection to mice compared to 3G11, which could be related to the observation that 3G11 interacts with bud-cells (see Fig 4), whereas 5H5 could target mother cells, the main infective propagules. Immunolabelling and growth inhibition assay with A. fumigatus suggests that 3G11 and 5H5 are more specific for linear and branched β-(1→3)-glucans, respectively. Linear glucans are exposed on the germ tube, and during growth, neo-synthesis and branching of β-(1→3)-glucan, the two essential phenomena [38], are targeted by 3G11 and 5H5, respectively.

### Conclusions

Several examples of anti-fungal antibodies with protective efficacy have been described [28,39–43]. Natural polysaccharides purified from fungal cells where been used to induce anti-fungal antibodies. In this study, for the first time a synthetic oligosaccharide derivative was used successfully for the development of mAbs with protective efficacy. β-Glucan is the major component of the fungal cell wall; we developed monoclonal anti-β-(1→3)-D-glucan antibodies by immunization of mice with a BSA-conjugate of synthetic nona-β-(1→3)-D-glucoside and hybridoma technology. The carbohydrate affinity of mAbs 3G11 and 5H5 was assessed by SPR and was approximately 19 nM and 1.9 nM, respectively. In addition, the carbohydrate specificity of the mAbs was determined using a thematic glycoarray built from a series of synthetic oligosaccharide ligands structurally related to the characteristic β-(1→3)-D-glucan fragments. Immunolabelling studies confirmed the selectivity of developed mAbs in detecting β-(1→3)-D-glucan on the surface of the fungal cell wall. Further, these antibodies could inhibit fungal growth *in vitro*, facilitate fungal phagocytosis by host immune cells *in situ*, and showed efficacy in combination therapy by decreasing the required drug concentration of fluconazole compared to monotherapy. Moreover, clear protective efficacy was observed for both mAbs in *in vivo* experiments using a lethal mouse model of systemic candidiasis.

## Supporting information

S1 File Immunofluorescence microscopy of different microbial species. Immunolabelling for *C*. *tropicalis*, *C*. *parapsilosis*, *C*. *dubliniensis*, *D*. *hansenii*, gram-positive (*B*. *infantis*, *B*. *longum*, *E*. *faecalis*, *L*. *plantarum*, and *S*. *aureus*) and gram-negative (*A*. *faecalis*, *P*. *mirabilis*, *P*. *aeruginosa*, and *S*. *enterica*) bacterial species by mAbs 3G11 and 5H5.
(PDF)

S1 Fig Assaying of bacterial supernatants with mAbs 3G11 and 5H5. Sandwich enzyme-linked immunosorbent assay (ELISA) with anti-**G9** antibodies: the wells of microtiter plates were coated with 200 ng mAb 3G11 (**A**) or mAb 5H5 (**B**) and incubated with serially diluted culture supernatants of indicated bacterial cultures; horseradish peroxidase-conjugated mAb 5H5 was used for sandwich ELISA.
(PDF)
