# Supplementary material for: Novel mouse monoclonal antibodies specifically recognizing β-(1→3)-D-glucan antigen
Source: PLoS One. 2019 Apr 25;14(4):e0215535. doi: 10.1371/journal.pone.0215535 (PMC6483564; doi:10.1371/journal.pone.0215535)

Candida tropicalis

3G11

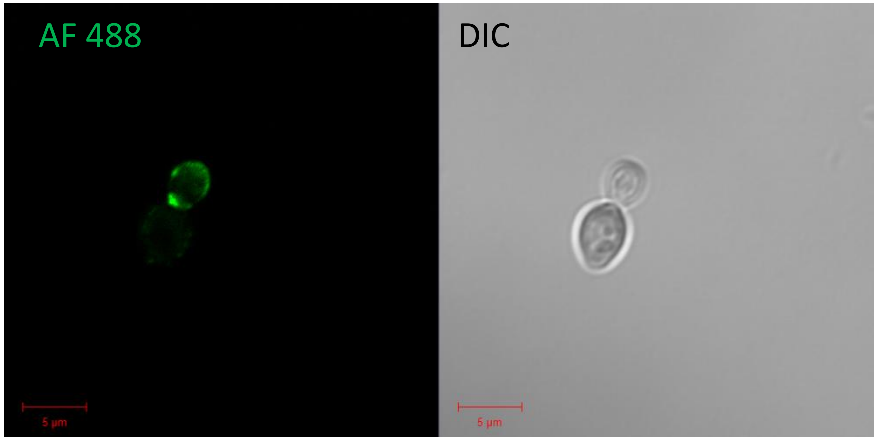

5H5

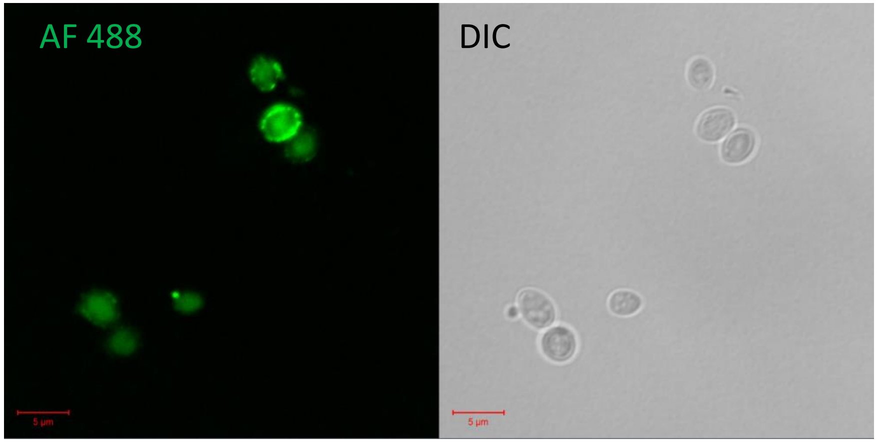

Candida parapsilosis

3G11

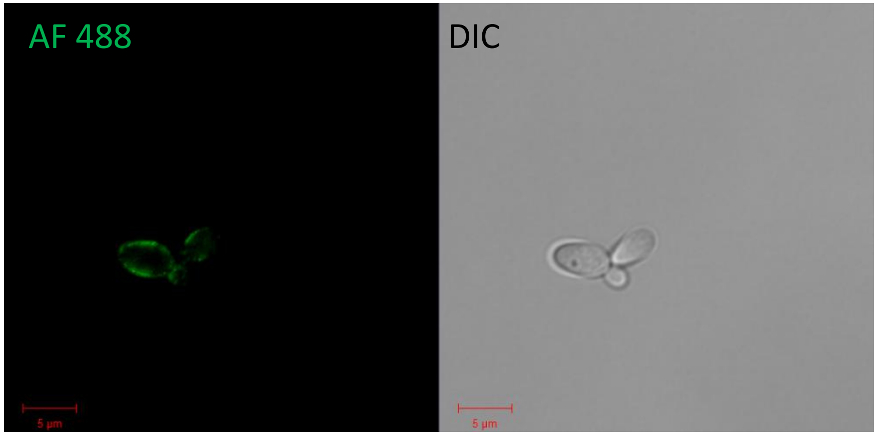

5H5

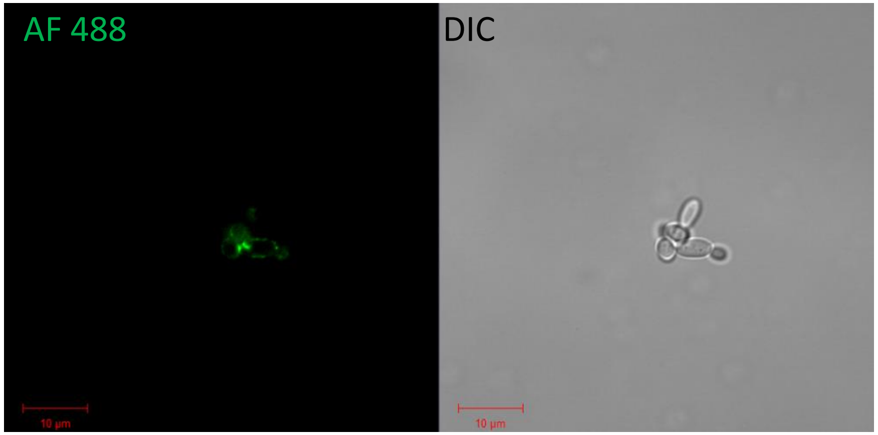

Candida dubleniensis

3G11

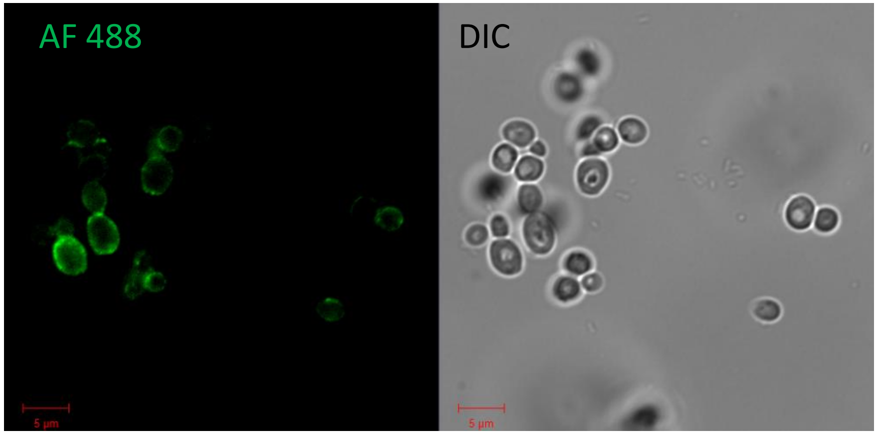

5H5

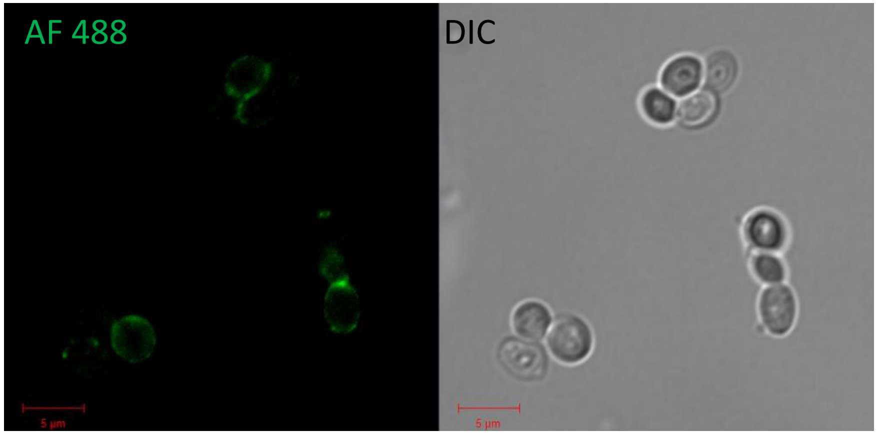

Debaryomyces hansenii

3G11

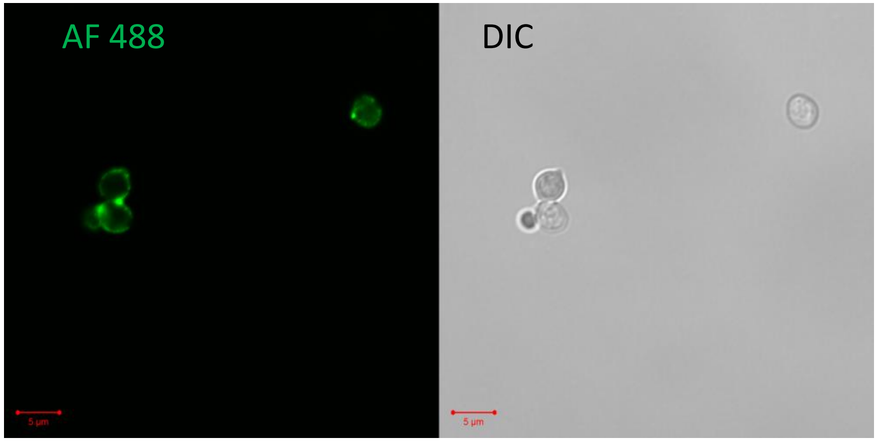

5H5

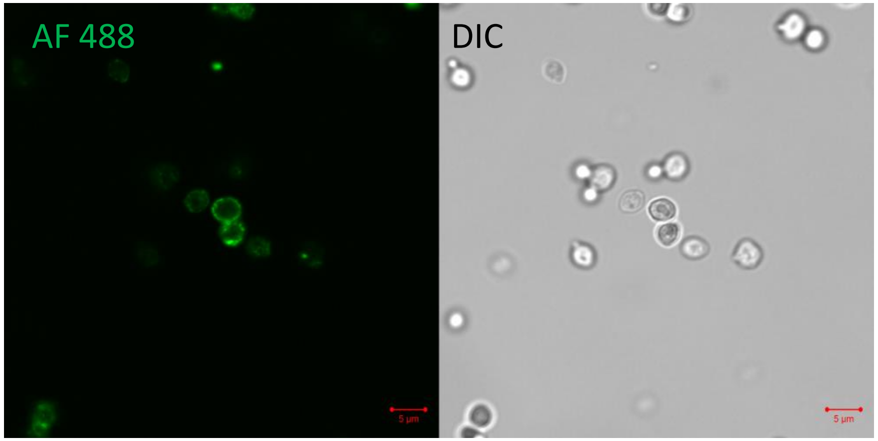

Bifidoabterium infantis

3G11

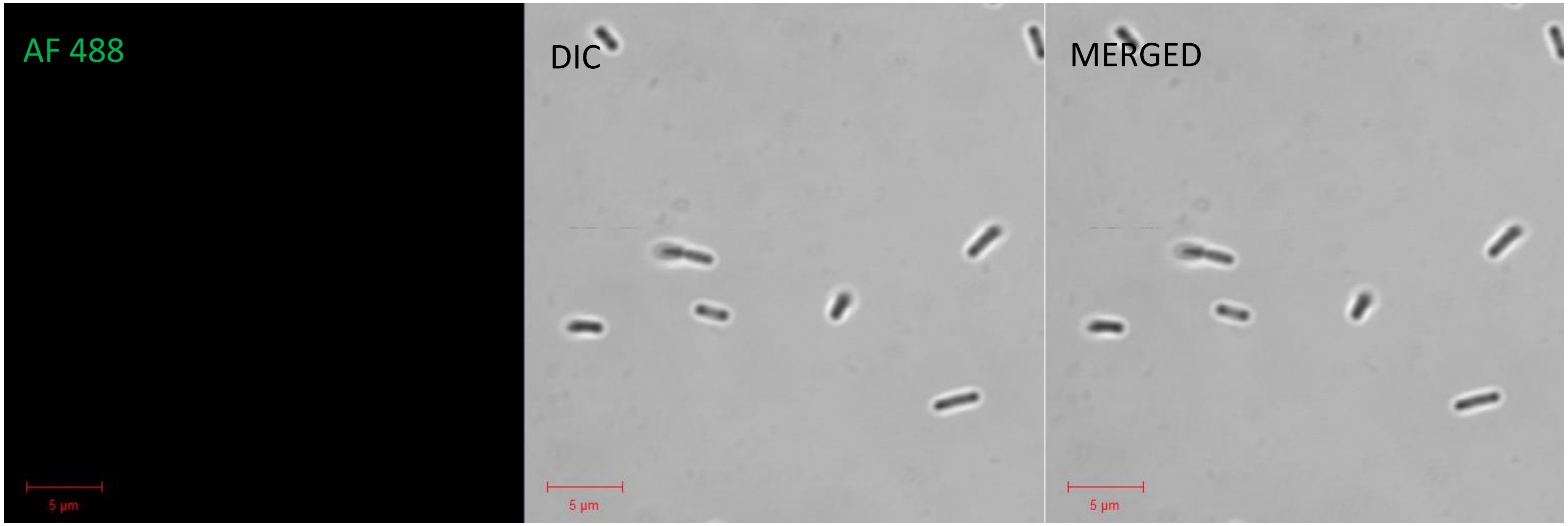

5H5

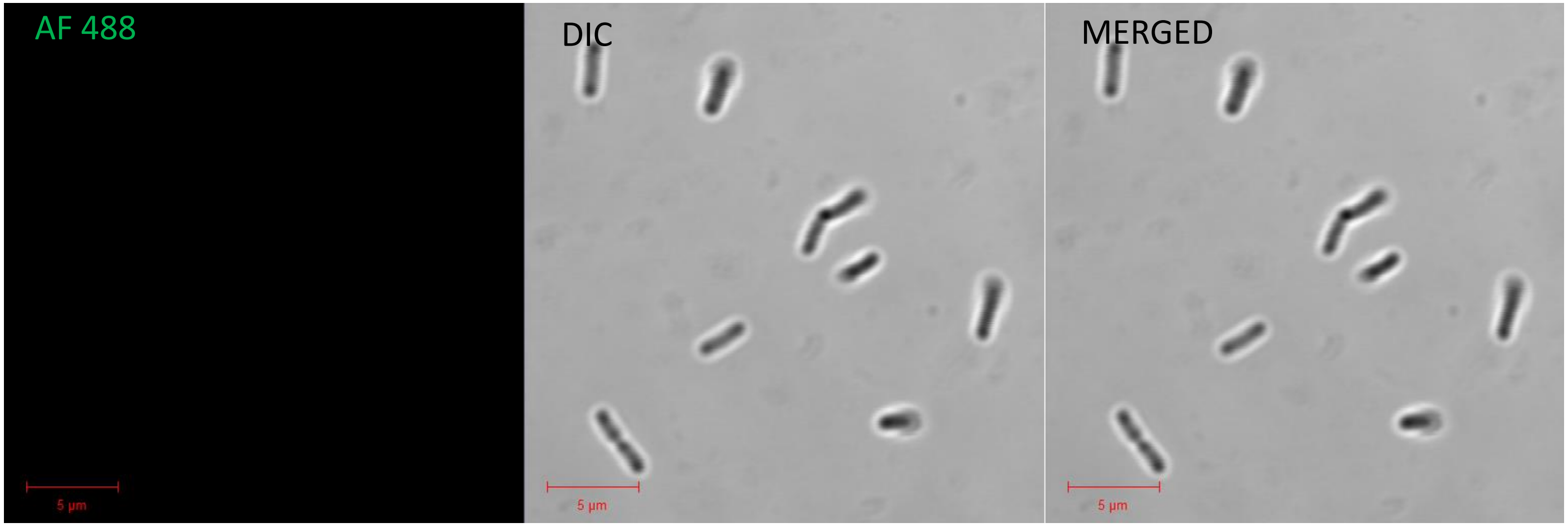

Bifidoabterium Longum

3G11

5H5

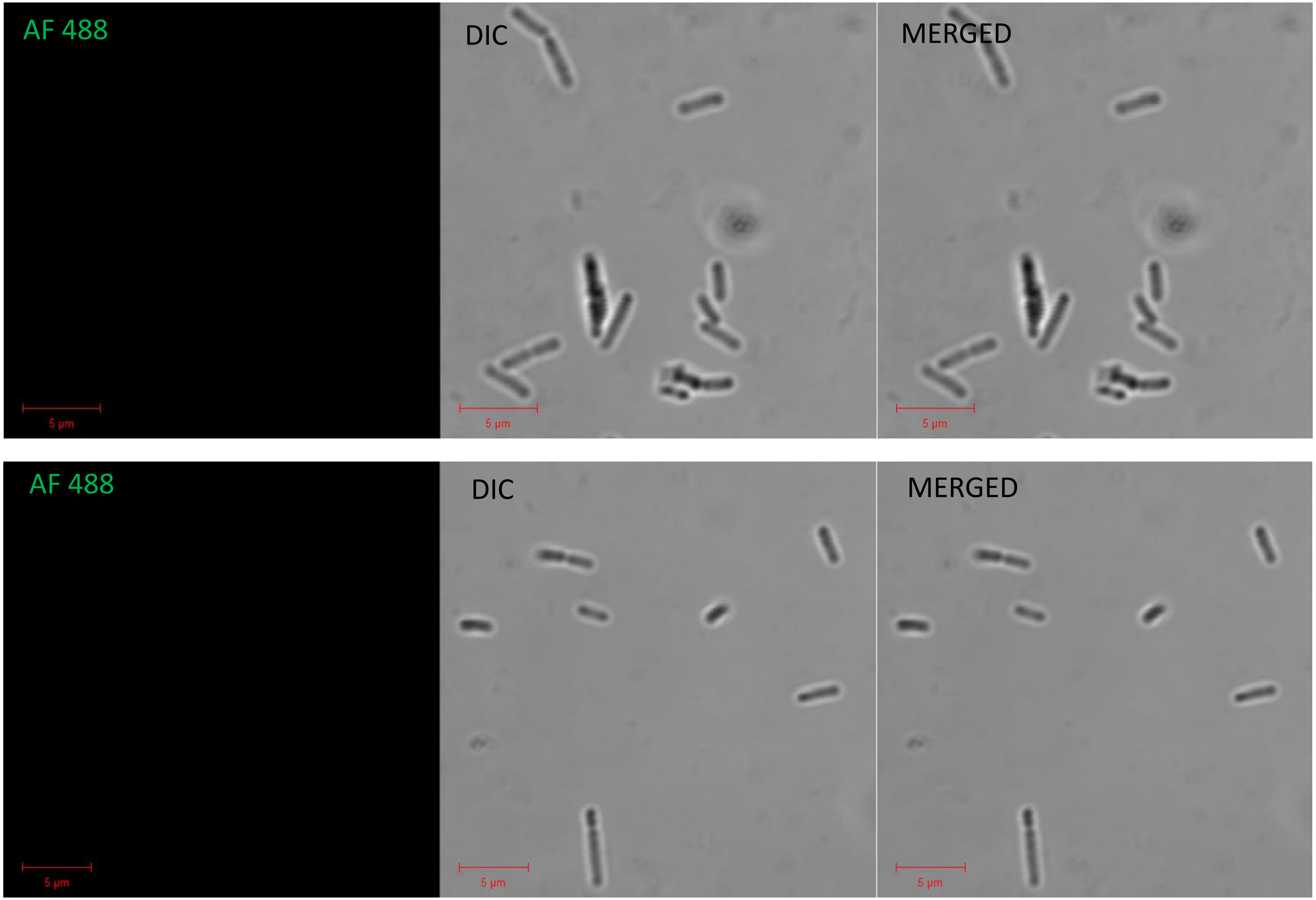

Enterococcus faecalis

3G11

5H5

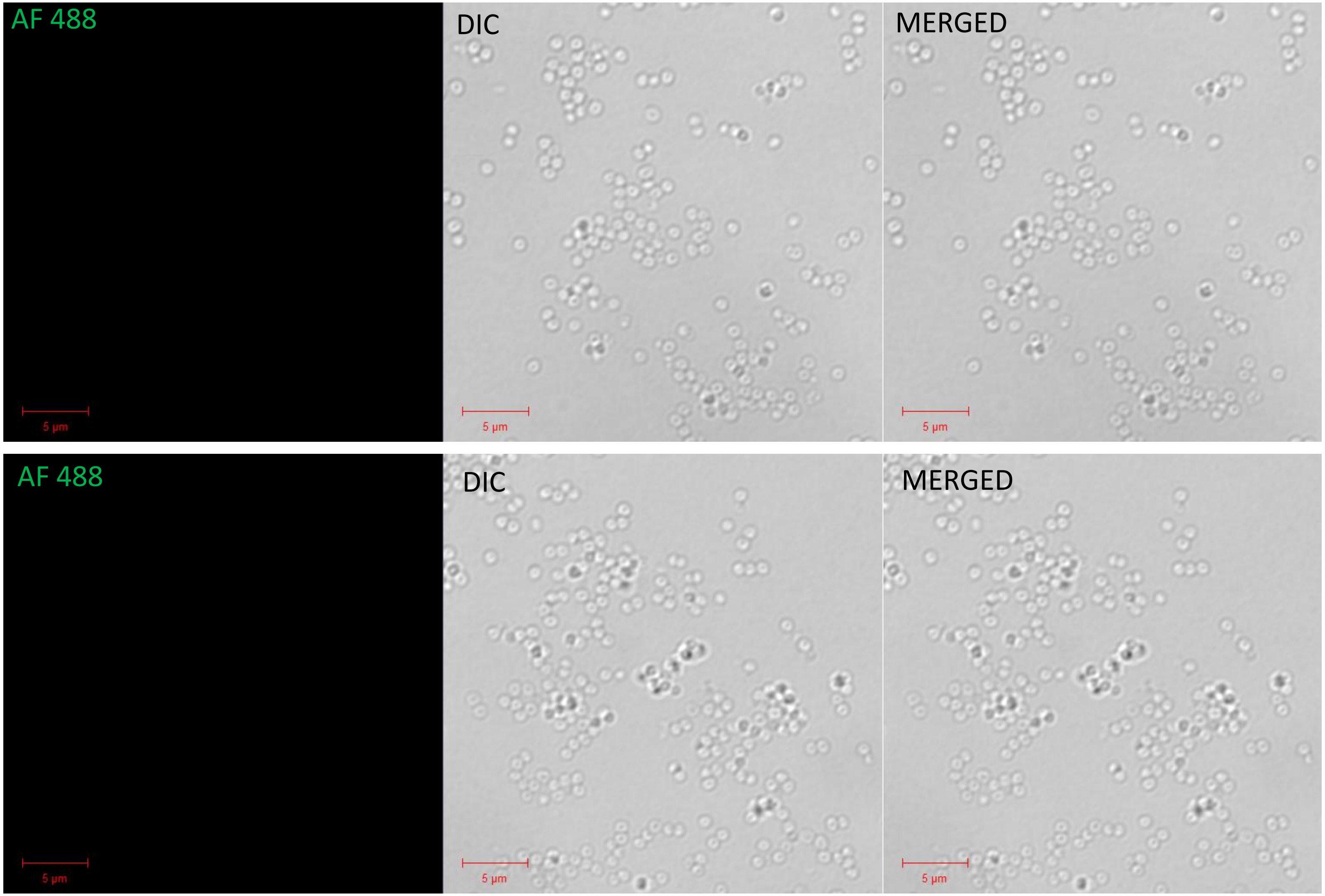

Lactobacillus plantarum

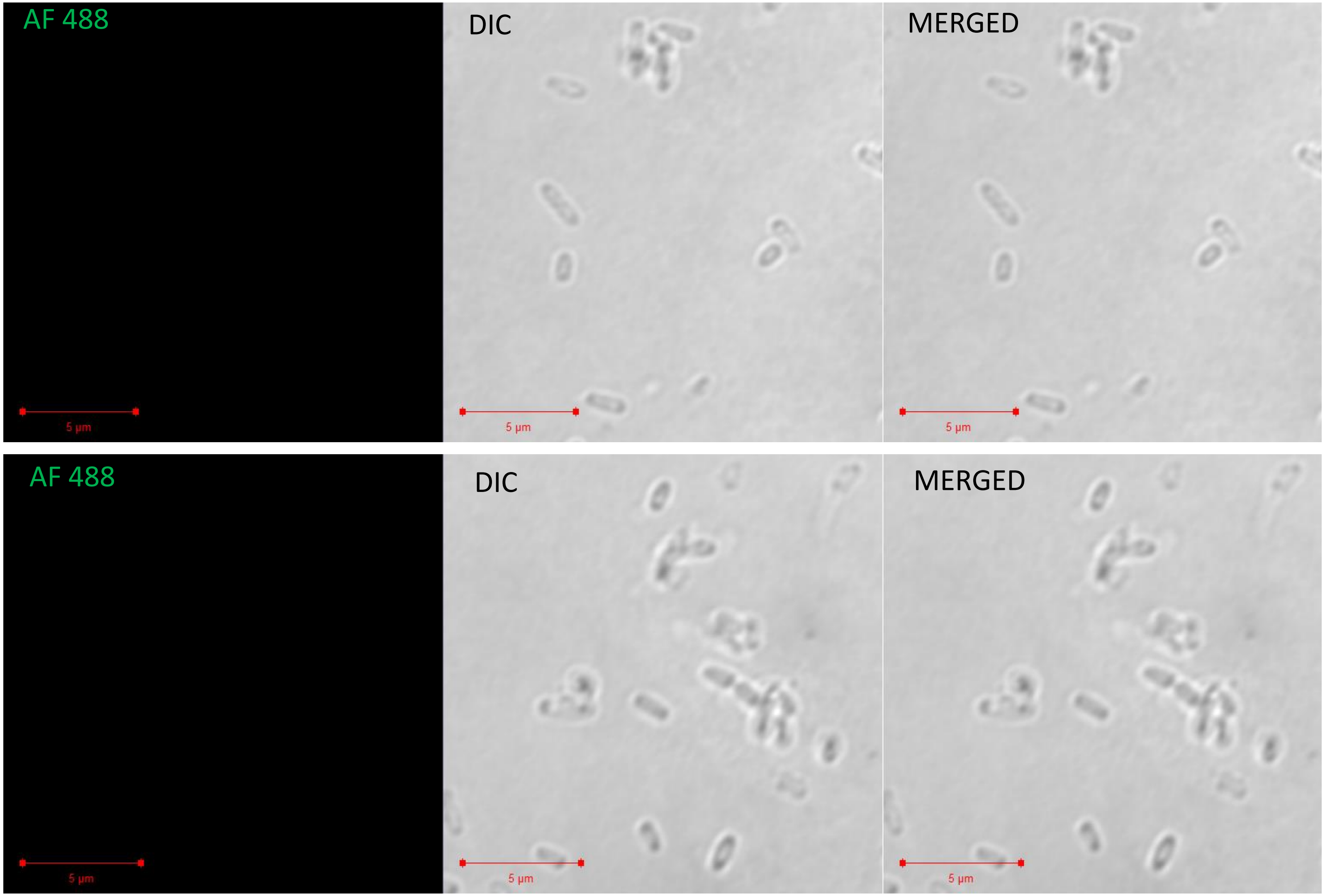

3G11

5H5

Staphylococcus aureus

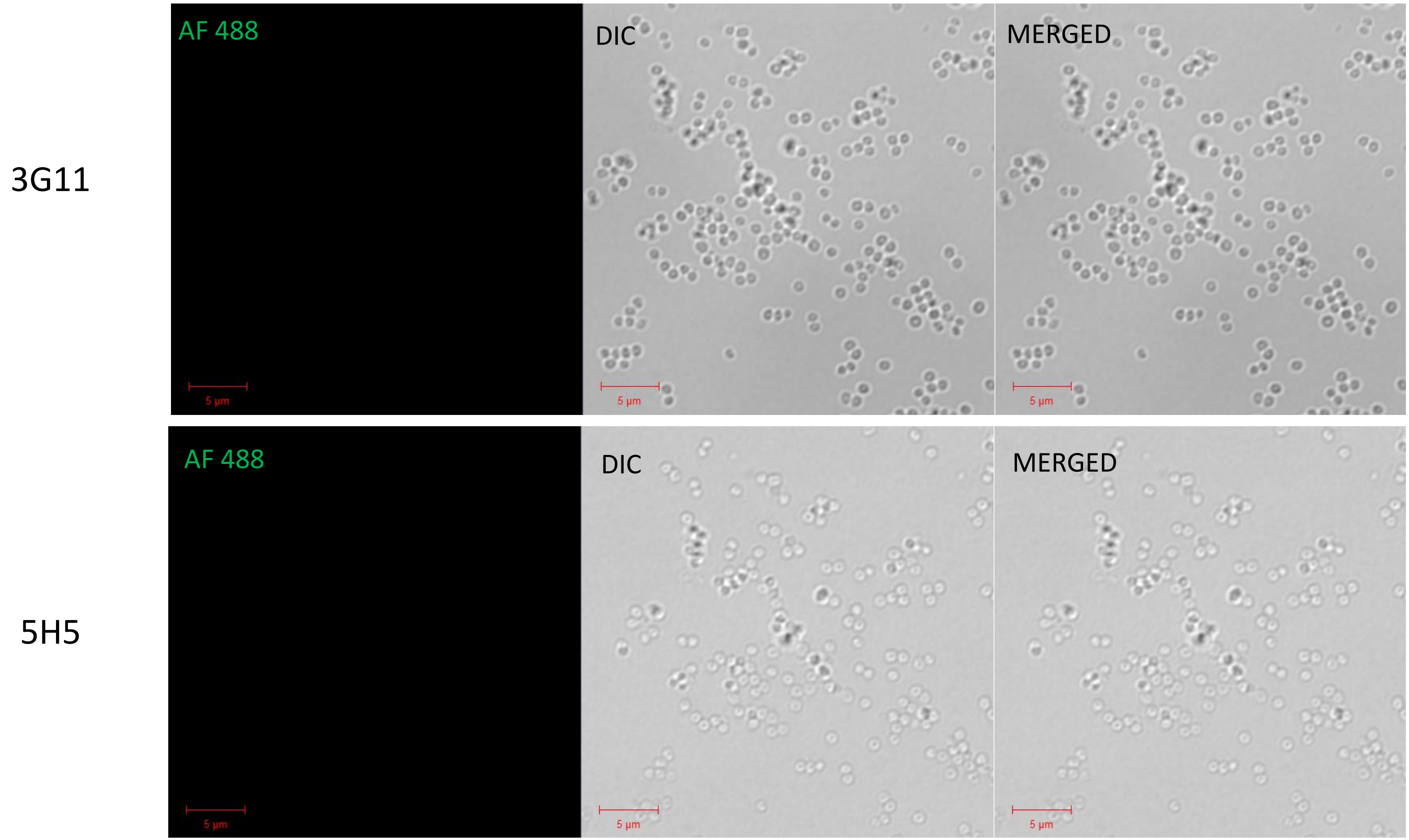

Alcaligenes faecalis

3G11

5H5

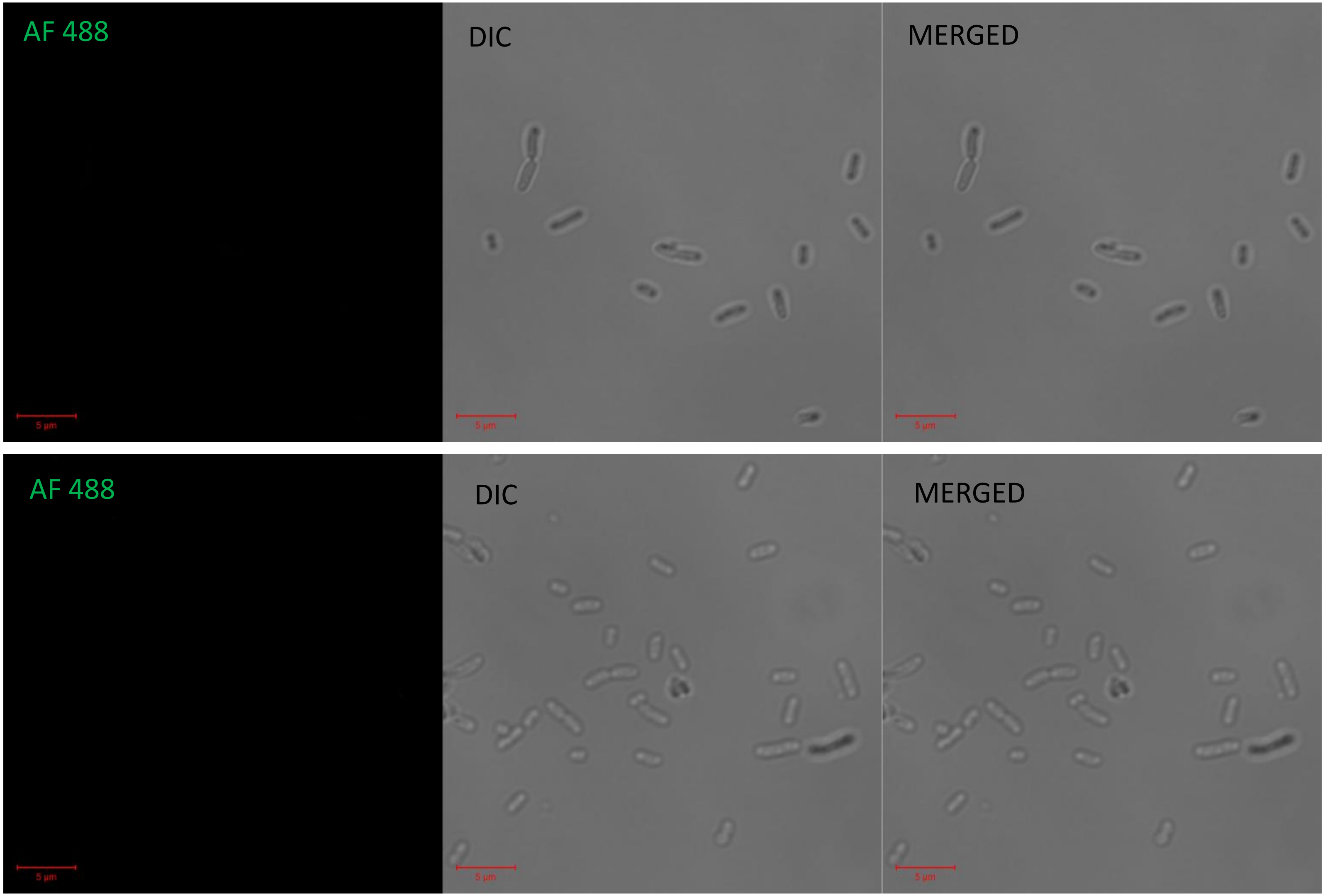

Proteus mirabilis

3G11

5H5

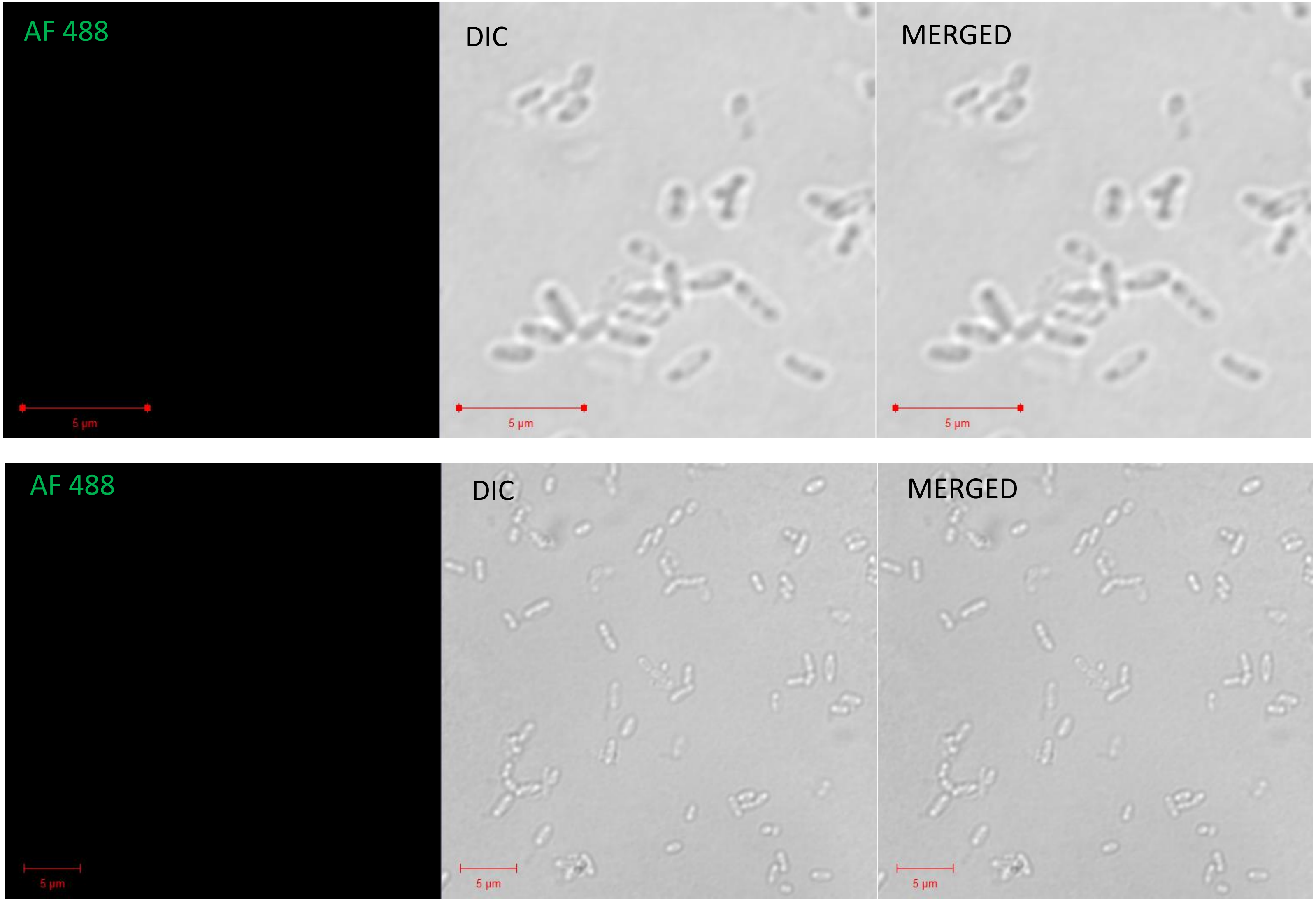

Pseudomonas aeruginosa

3G11

5H5

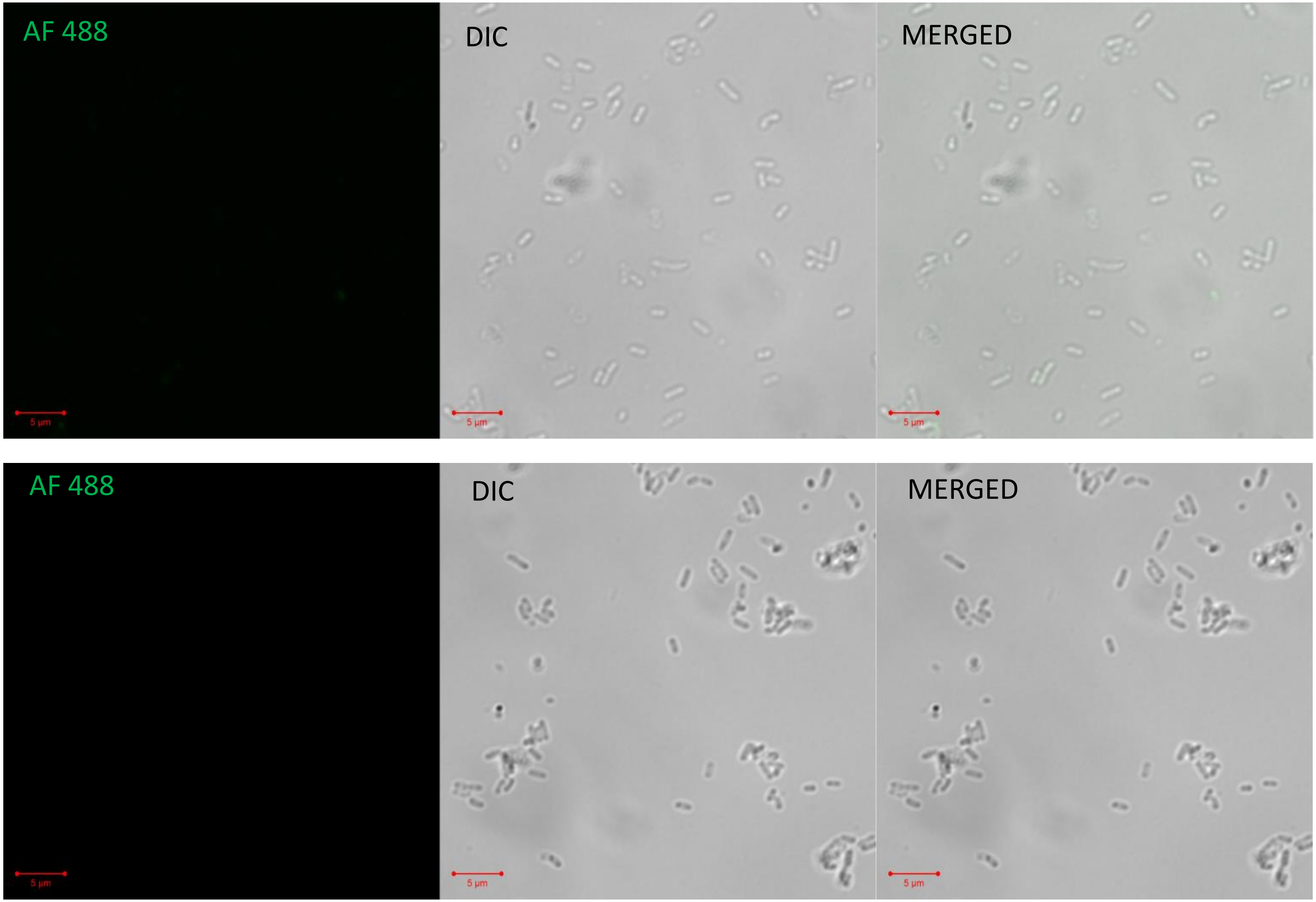

Salmonella enterica

3G11

5H5

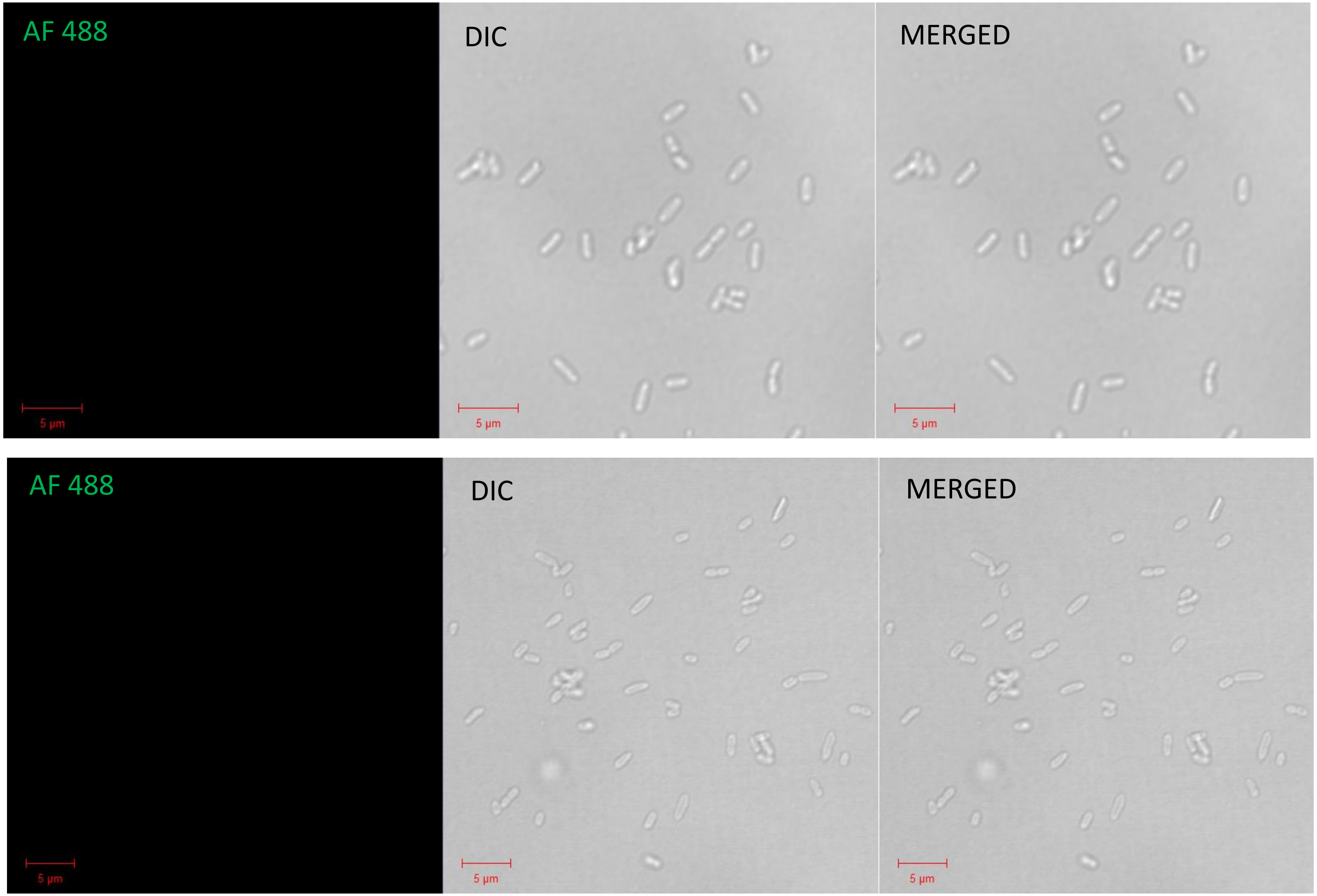

Supplement: S1 File — Immunolabelling for C. tropicalis, C. parapsilosis, C. dubliniensis, D. hansenii, gram-positive (B. infantis, B. longum, E. faecalis, L. plantarum, and S. aureus) and gram-negative (A. faecalis, P. mirabilis, P. aeruginosa, and S. enterica) bacterial species by mAbs 3G11 and 5H5. (PDF) [file pone.0215535.s001.pdf]
