# Supplementary figures and images for: Novel mouse monoclonal antibodies specifically recognizing β-(1→3)-D-glucan antigen
Source: PLoS One. 2019 Apr 25;14(4):e0215535. doi: 10.1371/journal.pone.0215535 (PMC6483564; doi:10.1371/journal.pone.0215535)

**A**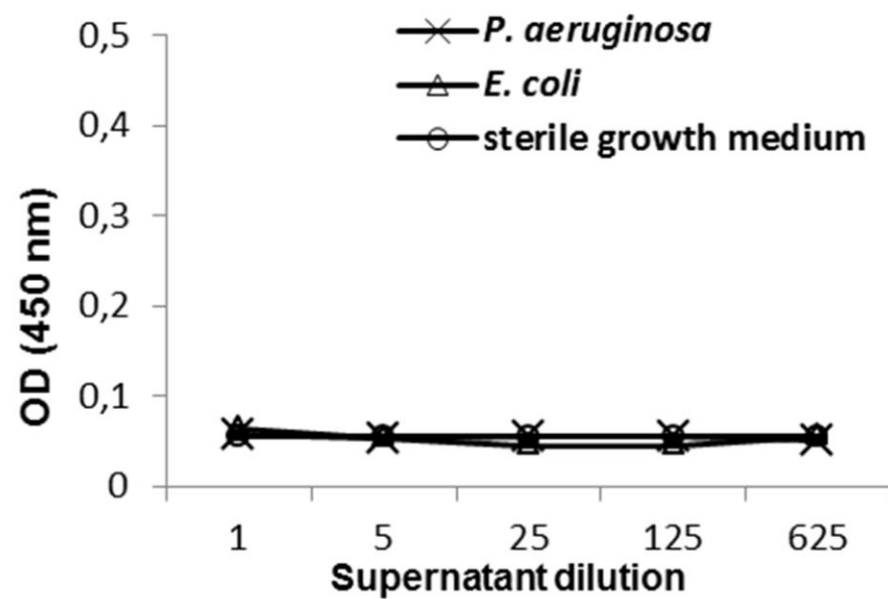**B**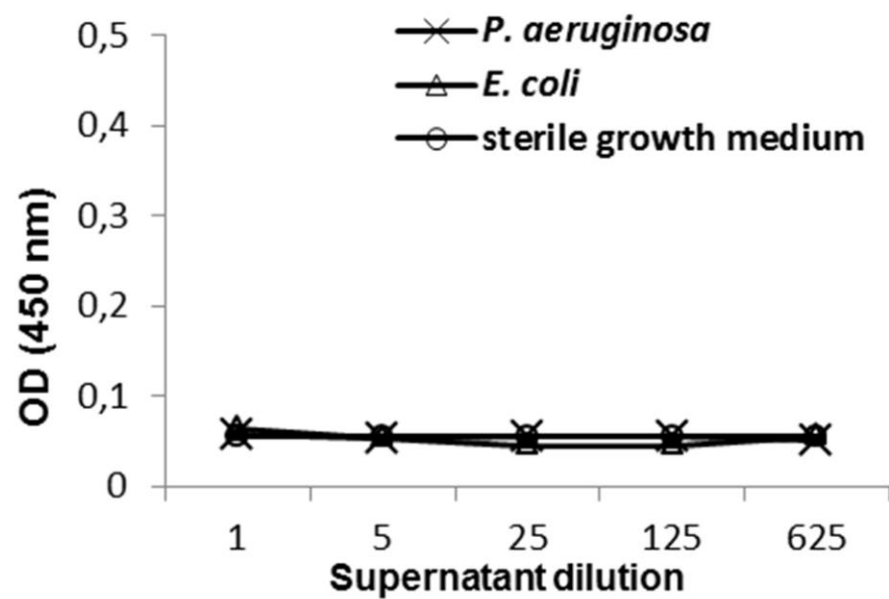

Supplement: S1 Fig — Sandwich enzyme-linked immunosorbent assay (ELISA) with anti-G9 antibodies: the wells of microtiter plates were coated with 200 ng mAb 3G11 (A) or mAb 5H5 (B) and incubated with serially diluted culture supernatants of indicated bacterial cultures; horseradish peroxidase-conjugated mAb 5H5 was used for sandwich ELISA. (PDF) [file pone.0215535.s002.pdf]
